# Multisite regulation integrates multimodal context in sensory circuits to control persistent behavioral states in *C. elegans*

**DOI:** 10.1038/s41467-023-38685-1

**Published:** 2023-05-26

**Authors:** Saurabh Thapliyal, Isabel Beets, Dominique A. Glauser

**Affiliations:** 1 Department of Biology, University of Fribourg, 1700 Fribourg, Switzerland; 2 Neural Signaling and Circuit Plasticity Group, Department of Biology, KU Leuven, 3000 Leuven, Belgium

**Keywords:** Sensory processing, Cellular neuroscience, Molecular neuroscience

## Abstract

Maintaining or shifting between behavioral states according to context is essential for animals to implement fitness-promoting strategies. How the integration of internal state, past experience and sensory inputs orchestrates persistent multidimensional behavioral changes remains poorly understood. Here, we show that *C. elegans* integrates environmental temperature and food availability over different timescales to engage in persistent dwelling, scanning, global or glocal search strategies matching thermoregulatory and feeding needs. Transition between states, in each case, involves regulating multiple processes including AFD or FLP tonic sensory neurons activity, neuropeptide expression and downstream circuit responsiveness. State-specific FLP-6 or FLP-5 neuropeptide signaling acts on a distributed set of inhibitory GPCR(s) to promote scanning or glocal search, respectively, bypassing dopamine and glutamate-dependent behavioral state control. Integration of multimodal context via multisite regulation in sensory circuits might represent a conserved regulatory logic for a flexible prioritization on the valence of multiple inputs when operating persistent behavioral state transitions.

## Abstract (web summary)

Animals must integrate multiple inputs to switch between behavioral states. Here, authors discover two *C. elegans* behavioral states and reveal a common regulatory logic through which they are controlled according to multimodal environmental changes.

## Introduction

Animals continuously integrate information from external environment with their past experience and internal states to generate adaptive behavioral states. This context-dependent modification of behavioral states in turn impacts sensory processing, sensory integration and animal’s response to the environment^1–3^. Animals show transient or persistent modifications in their behavioral state based on shift in integrated valence of the context to ensure survival and maximize their fitness^4–6^. E.g., animals will use multiple sources of information in order to select between different foraging strategies balancing the risks and benefits of local resource exploitation versus long-range exploration. Failure to execute behavioral state transitions will cause reduced behavioral flexibility, which can impact ecological performance, alter physiology and which is a hallmark of many human pathologies such as autism spectrum disorders and mood disorders^7–10^. Recently, several studies in mammals, vertebrates and fruit fly have highlighted functions and mechanisms of transition to distinct transient or persistent behavioral states^11–13^. However, several questions still remain largely unanswered. (i) How past and current experience of a single cue integrates with context to trigger behavioral state transitions that may persist for hours? (ii) How continuous integration of state-specific signals coordinate multi-dimensional behavioral responses to generate sophisticated and coherent navigation strategies? The size and complexity of the nervous system represent major challenges for the integrative studies needed to understand the underlying circuit, cellular and molecular mechanisms.

*C. elegans* has become a popular animal model to understand behavioral state transitions with tools and techniques available to gain multi-layered mechanistic insights (see^14^, for a review). Previous studies have identified multiple behavioral states in *C. elegans* based on differential locomotion, egg laying, or mate search and regulated by diverse sensory and physiological cues. In the presence of food, worms show roaming and dwelling states, whereas without food, execute local or global search^15–19^. Each of these behavioral states is characterized by a specific locomotory pattern in order to promote a specific exploitation/exploration strategy. E.g., dwelling animals move slowly and produce head swipes while pumping food (favoring resource exploitation), whereas animals in global search combine elevated speed and infrequent turns to increase dispersal (favoring exploration to find new resources). Egg laying impacts reproductive fitness and worms display temporal variations in bouts of egg laying activity, which are impacted by feeding status and other sensory cues^20,21^. Sleep and wakefulness are one of the vastly studied behavioral states. Similar to mammals, worms also display sleep-like behavior as ‘lethargus’ during larval stage transitions and ‘sickness-induced sleep’ in response to external physiological stress^22,23^. Transitions between these diverse behavioral states in response to various environmental cues ensure cellular homeostasis with physiological and reproductive fitness^24^.

Behavioral states and transitions are characterized based on multi-dimensional phenotypic differences. These are readily quantified with machine-vision-based approach in *C. elegans*, capturing behavioral nuances which are difficult to detect by manual examination^25–27^. Moreover, neuron activity recording in vivo and manipulation tools allowing mechanistic dissection at circuit levels can link distinct neuronal activity patterns with different behavioral states^28–32^. Genetic analyses in different studies revealed the role of conserved neuromodulatory pathways as dopamine, serotonin, tyramine, octopamine and neuropeptide signaling in mediating behavioral state transitions^15,33–36^.

*C. elegans* can sense and modulate behavioral states in response to a wide range of sensory and physiological cues such as touch, odors, light, sound, oxygen, CO_2_, and temperature from the environment^37–42^. For our study, we chose temperature as sensory/physiological cue because past and current thermosensory sensory experience can be precisely controlled by varying cultivation temperature and performing temperature shifts. *C. elegans* senses a wide range of temperature, and execute thermotaxis to stay at a preferred temperature in the ‘innocuous range’ of 13–25 °C, while avoiding extreme ‘noxious’ temperatures^43–45^. Based on context, distinct sensory neurons encode temperature information in phasic and tonic neuronal responses and generate appropriate behavioral outputs^46^. Whereas short-lasting processes (second–minutes timescale) underlying temperature-dependent navigation in spatial thermogradient has been extensively studied, we know surprisingly little on temperature-dependent persistent behavioral states and transitions over longer timescales.

In addition to previously described dwelling, local search and global search states, we demonstrate here that temperature history and current environmental temperature interact with feeding status to drive animals in additional behavioral states (which we propose to call scanning and glocal search) with unique thermoregulatory and foraging benefits. Each state is controlled by specific neuropeptide-based signaling from two separate tonic thermosensory pathways. In order to compute contextual inputs over time and modalities, a similar logic is used in each pathway, relying on the concerted regulation of multiple processes, which occurs only when a specific combination of environmental/internal signals are achieved. Given the conservation of the molecular players involved, we speculate that multimodal context processing via coordinated multi-site regulation along tonic sensory pathways might be a widespread regulatory solution mediating persistent behavioral transitions in other species or sensory modalities and that its malfunction could underlie human behavioral flexibility disorders.

## Results

### An analytic framework for dissecting temperature and food-dependent behavioral states and transitions

**Fig. 1:**
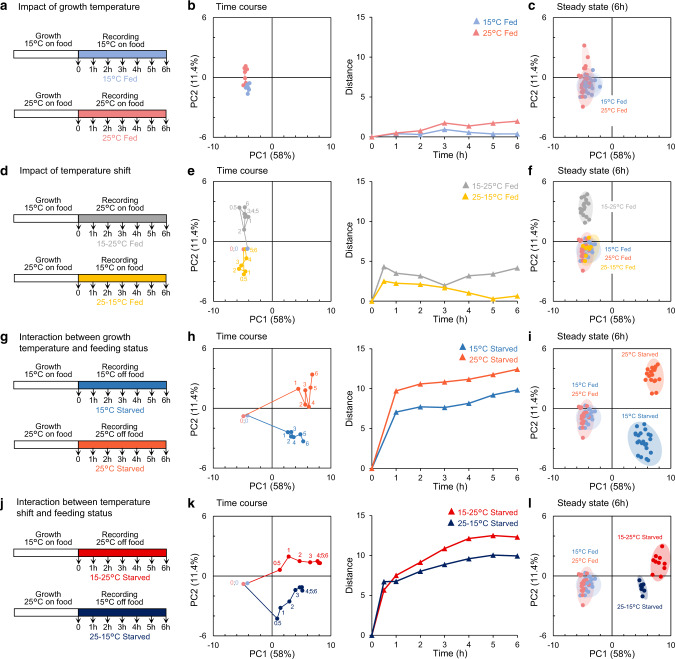
Current and past temperature interact with food availability to set worms in distinct behavioral states. Behavioral states of young adult *C. elegans* exposed to defined thermal and feeding treatments (as depicted in **a,** **d,** **g** and **j**) presented as projections over the two main PCA components from a single analysis of postural and motion parameters over all conditions (**b,** **c,** **e,** **f,** **h,** **i,** **k,** **l**). Behavioral transitions unfolding over 6 h presented (i) as its evolution in this PCA space (left panels in **b,** **e,** **h,** **k**, with numbers indicating the post treatment time in h) and (ii) as a time courses of the distance between each time point and the starting point (*t* = 0) (right panels). Steady states reached after 6 h of the indicated thermal and/or feeding shifts (**c,** **f,** **i,** **l**). Positions of individual replicate average as data marks, 95% CI as colored ellipses. Each replicate as a separate worm population with ≥40 animals. Source data are provided as a Source Data file.

To investigate how multimodal context is processed to orchestrate behavioral state transitions and their maintenance, we systematically analyzed the behavior of *C. elegans* while varying thermosensory history (growth temperature at 15 versus 25 °C), current thermosensory inputs (with thermal shifts from 15 to 25 or from 25 to 15 °C) and food availability (in fed versus starved animals). We recorded behavioral state snapshots in isothermal environments (Supplementary Fig. 1a, b) and compared multiple timepoints to highlight behavioral transitions and persistent states. We then performed a detailed quantification of *C. elegans* posture and locomotion, focusing on a set of 47 interpretable behavioral parameters (Supplementary Fig. 1c and Supplementary Data 1), and visualized behavioral states in a common multiparametric space from a single Principal component analysis (PCA) gathering all the conditions examined in the present study for wild type (Fig. 1a, d, g, j). Below, we sequentially describe the impact of long-term growth temperature, of current thermosensory inputs and their interactions with food availability.

### Worms on food are in a dwelling state regardless of their growth temperature

**Fig. 2:**
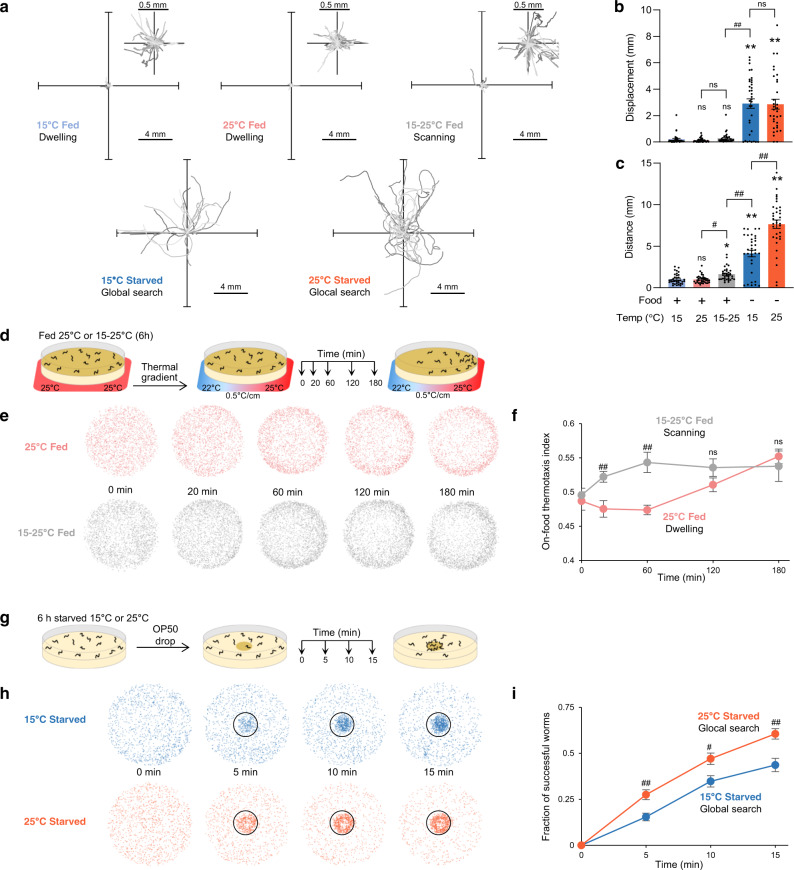
Food and temperature-dependent behavioral states underly different navigation strategies with different food seeking and thermoregulatory performances. One-minute worm trajectories recorded in isothermal environments (35 for each condition) and plotted from a single starting (0, 0) coordinate (**a**). Enlarged representation for worms in dwelling and scanning (inset). Average ± s.e.m. and individual data points for animal displacement (**b**), corresponding to how far animals moved from their starting point and covered distance (**c**), corresponding to the path length. *n* = 30 animals. *p < 0.05 and **p < 0.01 versus 15 °C Fed condition, #*p* < 0.05 and ##, *p* < 0.01 versus the indicated control by Bonferroni posthoc tests. On-food thermotaxis assay in fed animals revealing a faster thermotactic movement toward recent growth temperature in scanning animals (6 h after warming) as compared to dwelling animals held at 25 °C (**d**, **e**, **f**). Schematic of the assay unfolding (**d**), overlayed worm positions over multiple assays (**e**), on-food thermotaxis index time course (average ± s.e.m., **f**). *n* = 10 assays per condition. Food drop assay in which starved animals in glocal search mode at 25 °C perform better than animals in global search mode at 15 °C (**g**, **h**, **i**). Schematic of the assay unfolding (**g**), overlayed worm positions over multiple assays (**h**), time course of the fraction of successful worms (average ± s.e.m., *n* = 9 independent assays, (**i**)). ##*p* < 0.01 versus the other condition at the same time point, by Bonferroni posthoc tests. Source data are provided as a Source Data file.

To address the impact of growth temperature, we recorded behavioral snapshots over 6 h of animals cultivated at 15 or 25°C without changing their temperature (Fig. 1a). We found that animals at different temperatures on food show very similar behavioral states (Lower left quadrant in PCA space, Fig. 1b–c and Supplementary Fig. 2). This trend was also confirmed when we separately analyzed the motion and the posture of the animals in two separate PCA analyses (Supplementary Fig. 3 and 4). This common behavioral state corresponds to the previously described dwelling state^15,16,19,47^, where animals move slowly and spend most of their time with paused locomotion to feed (Fig. 2a, Supplementary Fig. 5 and Supplementary Movie 1). Hence, fed animals maintained on food at a constant temperature are in a similar dwelling state regardless of their growth temperature.

### Cooling transiently reinforces dwelling, while warming promotes a persistent scanning state

Next, to understand how current thermosensory inputs impact behavioral states, we analyzed the behavior of animals grown at 25 °C and shifted to 15 °C at the onset of the recordings for 6 h (Fig. 1d). In response to cooling, animals transiently transitioned to a reinforced dwelling state (deeper into the PCA lower left quadrant, Fig. 1e–f), with decreased forward and backward locomotion frequency and concomitantly increased pausing duration and foraging amplitude (Supplementary Fig. 5).

**Fig. 3:**
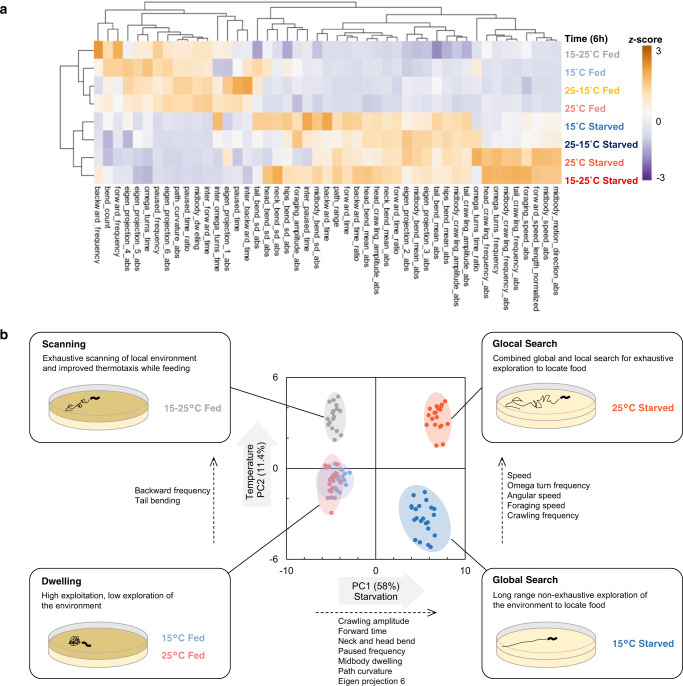
Distinct food and temperature-dependent behavioral codes underlie specific behavioral exploitation/exploration strategies. Behavioral steady-states after 6 h in the indicated food and temperature-dependent condition (corresponding to the treatments depicted in Fig. 1a, d, g, j). Heat-map of behavioral parameters (*z*-scores) across the indicated conditions and hierarchical clustering trees based on Euclidian distances (**a**). Behavioral state representation as in Fig. 1c, f, i with subjective behavioral state interpretation annotations and illustration of the corresponding exploitation/exploration strategies (**b**). Source data are provided as a Source Data file.

Next, we conducted the reverse experiment and examined the impact of warming, by shifting the animals from 15 to 25 °C (Fig. 1d). Animals transitioned to a new long-lasting steady state (shift toward the PCA upper left quadrant, Fig. 1e–f, Supplementary Movie 1). Warming modulated both postural (with e.g., reduced tail bending) and motion aspects (Fig. 3a, Supplementary Fig. 3–5). While keeping low speed, animals increased their foraging speed, reduced pausing time and persistently increased reversals (backward frequency, Supplementary Fig. 5) enabling animals to regularly change their direction. We examined the geometry of individual 1-min trajectories (Fig. 2a) and quantified the displacement of animals (distance from the start and the end of the path, Fig. 2b) and the total distance covered (cumulative distance along the path, Fig. 2c). As compared to animals held at 15 or 25 °C, animals shifted from 15 to 25 °C covered more distance, but kept the same low displacement values. Therefore, warming promotes a specific behavioral state, where animals frequently swap between forward and backward motion to more actively scan their local environment while feeding, without dispersing much faster. We will call this state: scanning.

What could be the advantage of the scanning state? We hypothesized that, in contrast to dwelling animals that barely move, scanning animals may better detect and respond to thermal gradients in order to thermotax while feeding. Indeed, it might be metabolically costly for worms to adapt their physiology to new temperature^48^ and beneficial to thermotax toward previous growth temperature as a thermoregulatory strategy^49^. We devised an on-food thermotaxis assay in which worms initially held in an isothermal environment at 25 °C are exposed to a smoothly appearing thermal gradient (0.5 °C/cm, 22–25 °C, Fig. 2d). While the magnitude of the distribution bias was more subtle than what can be observed in classical thermotaxis assays performed off-food^50^, both animals in dwelling (maintained at 25 °C) and animals in scanning (6 h after warming from 15 to 25 °C) moved back to 25 °C, but the latter drifted much faster (Fig. 2e, f and Supplementary Fig. 6).

In summary, shifting worms from 15 to 25 °C on food causes them to switch from a dwelling to a scanning state (Supplementary Movie 1), in which worms adopt a distinct posture and produce short-range movements linked to better thermotactic performances and thermoregulation (Fig. 3b).

### Starved animals engage in global search at 15 °C but glocal search at 25 °C

To examine the joint impact of animal feeding status and temperature, we recorded the behavior of starved animals cultivated at 15 and 25 °C. In line with the well-documented food deprivation-evoked shift from dwelling to food-search navigation mode^17,18,51^, food-deprivation at either temperature caused persistent behavioral changes, affecting both postural and motion parameters (Figs. 1g–i, 3a, Supplementary Fig. 3–4) and causing a right shift in our PCA space (with increasing PC1 values, Fig. 3b). Regardless of temperature, food-deprivation produced a rapid decrease in pausing and dwelling, with concomitant increase in the time spent in forward locomotion, as well as a progressive increase in reversal duration and postural alterations (Supplementary Fig. 5), consistent with a previously described global search state^17,52,53^.

Very interestingly, however, current temperature had a strong impact to steer worms into distinct behavioral states at 15 or 25 °C (lower and upper right quadrants in PCA space, Fig. 1g–l, Supplementary Fig. 2). Distinctively after starvation at 25 °C, worms increased speed, foraging speed, angular speed, omega turn and crawling frequency and produced longer and more twisty trajectories, contrasting with the shallowly curving radial trajectories at 15 °C (Fig. 2a, and Supplementary Movie 2). The worms covered more distance at 25 °C compared to 15 °C, however the displacement at 25 °C was similar to that at 15 °C, indicating that the increased locomotory activity at 25 °C did not enhance dispersal (Fig. 2b–c). We obtained similar results with Monte-Carlo simulations emulating worm dispersal with empirically measured speed and turn frequency values (Supplementary Fig. 7a–c). These observations are consistent with a model in which starved worms at 15 °C are in a global search state and disperse fast to find food without exhaustively sampling the local environment along their path, whereas starved worms at 25 °C disperse equally fast, but simultaneously sample local environment (Fig. 2a–c and Supplementary Fig. 7a–c). Therefore, by markedly up-regulating speed and omega turn rates, starved worms at 25 °C are in a state enabling both local and global search, which we will call glocal search. Monte-Carlo stimulations separately controlling turning rate and speed confirmed the importance of a coordinated up-regulation of speed and turning rate to achieve the specific exploration pattern seen in the glocal search mode (Supplementary Fig. 8a–c). Interestingly, glocal search state is different from both previously described ‘local search’^14,17^ and ‘global search’^17,52,53^ states (Supplementary Fig. 9).

Intensive exploration of environment in glocal search mode (Supplementary Movie 2) seems energetically costly and would be expected to come with some compensatory ecological benefits. We hypothesized that animals in glocal search state would more efficiently detect and reach food sources in their surroundings. As an empirical test, we designed an assay in which a food drop was added onto a plate of foraging worms and their progression monitored (Fig. 2g). We found that animals in glocal search state at 25 °C performed better than animals in global search state at 15 °C (Fig. 2h–i).

Collectively, our data indicate that, based on current temperature, starved worms operate multidimensional behavioral changes to select between global or glocal search states, which tunes the efficacy and exhaustiveness of food-seeking exploration.

### Multimodal context is integrated to control behavioral state transitions

As a whole, the analyses reported so far provide a comprehensive picture of how food and temperature-associated contexts can steer worms into four distinct steady behavioral states (separate quadrants in our PCA analysis), each corresponding to an exploitation/exploration strategy with its respective advantages for environment sampling and navigation (Fig. 3b). Whereas in our PCA analysis, PC1 mostly reflects the impact of food availability and PC2 the impact of temperature, these two factors do not produce simple additive effects, but rather interact. On food, worms are in dwelling mode regardless of growth temperature, but after starvation, temperature has a very salient effect to select between global or glocal search. Furthermore, the recent thermal history is very important to trigger scanning entry only upon warming. Below, we dissect the cellular and molecular mechanisms controlling these specific behavioral states.

### Scanning is controlled by AFD and is linked to elevated resting calcium level

**Fig. 4:**
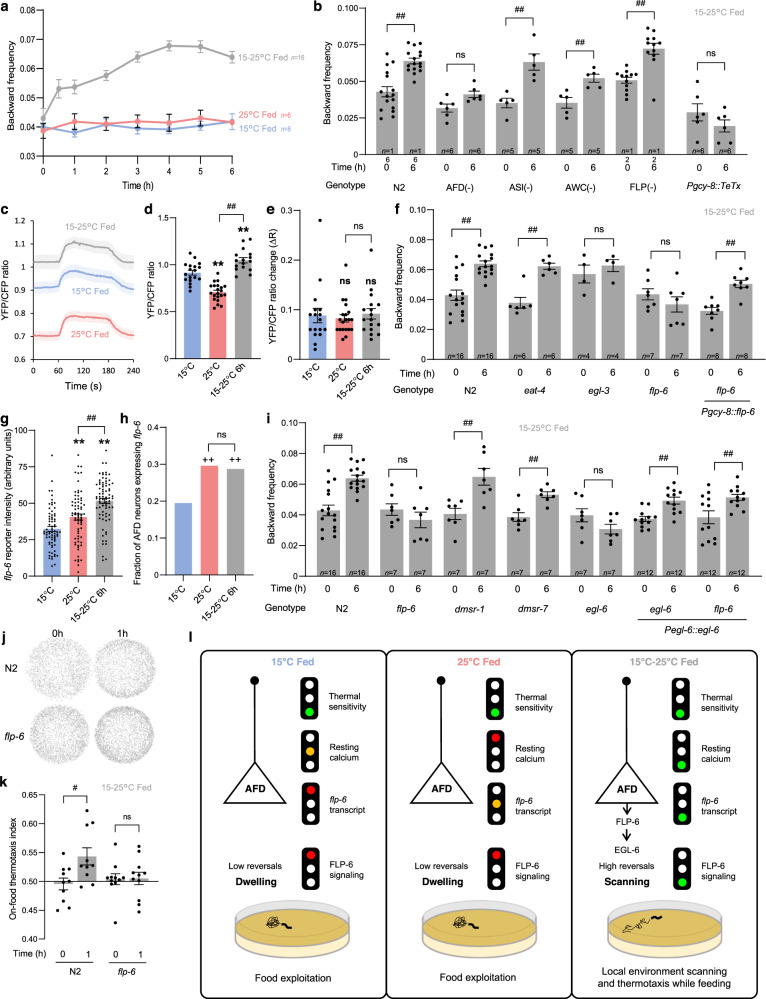
Multi-site regulation in AFD thermosensory neurons controls FLP-6-dependent scanning entry. Time course of worm backward frequency increase after warming from 15 to 25 °C (**a**). Backward frequency at *t* = 0 and *t* = 6 h following warming in the indicated genotypes (**b**, **f**, **i**); *Pgcy-8::TeTx*, transgene blocking synaptic transmission in AFD (**b**); *Pgcy-8::flp-6* transgene for AFD-specific *flp-6* rescue (**f**); *Pegl-6::egl-6*, transgene for *egl-6* over-expression and *flp-6* mutation by-pass analyses in *egl-6* and *flp-6* mutant background, respectively (**i**). Data as mean ± s.e.m.; indicated *n* correspond to independent assays, each scoring *≥*30 worms (**a**, **b**, **f**, **i**). Cameleon YFP/CFP ratio in AFD reporting the absolute intracellular calcium levels at rest and following a 2-min 10 °C thermal up-step in animals grown at 15 °C (*n* = 17 animals), grown at 25 °C (*n* = 20) or shifted from 15 to 25 °C for 6 h (*n* = 18) (**c**–**e**). Mean (±s.e.m.) traces (**c**), resting calcium levels (**d**) and temperature-evoked relative calcium changes (**e**). *flp-6* transcriptional reporter quantification comparing mean intensity ± s.e.m. (*n* = 69, 79, 79 neurons for 15°, 25° and 15–25°, resp., (**g**)) and fraction of AFD neurons with detectable signal (*n* = 354, 240, 275 neurons for 15°, 25° and 15–25°, resp., (**h**)). Result of on-food thermotaxis assays after 1 h (N2: *n* = 10; *flp-6*: *n* = 11 assays per condition) reported as in Fig. 2e-f, showing impaired temperature-dependent drift in *flp-6* mutants (**j**–**k**). **p < 0.01 versus 15 °C Fed condition, #*p* < 0.05 and ##*p* < 0.01 versus the indicated control by Bonferroni posthoc tests and ++*p* < 0.05 versus 15 °C Fed condition by Fisher’s exact test for contingency comparisons. ns not significant. Schematic of the multisite gating model controlling scanning entry (with gates as traffic lights, (**l**)). In dwelling animals, at least one gate is closed (red light), whereas they are all lifted (green lights) after warming to promote scanning entry. Source data are provided as a Source Data file.

To identify neurons required for warming-evoked transition to scanning on food, we genetically ablated candidate thermosensory neurons (AFD, FLP, AWC and ASI) and quantified backward frequency and tail bending, which are two hallmarks of scanning (Fig. 4a and Supplementary Fig. 10a). Changes in both parameters were still observed when ablating AWC, ASI or FLP, but strongly inhibited by the ablation of AFD (Fig. 4b and Supplementary Fig. 10b), suggesting primary role of AFD in the persistent behavioral switch to scanning. Blocking synaptic transmission from AFD using Tetanus toxin also abolished the backward frequency and tail bending modulation (Fig. 4b and Supplementary Fig. 10b), confirming the importance of neurotransmission from AFD for warming-evoked scanning entry.

AFD neurons respond to short-lasting thermosensory inputs with fast-adapting intracellular calcium transients, but can also encode thermal history as long-lasting changes in resting calcium levels^46,54,55^. To determine if warming-evoked scanning entry is associated with specific AFD calcium activity, we imaged AFD calcium levels using a ratiometric YC2.3 calcium sensor and compared animals grown at 15 °C, grown at 25 °C or exposed to warming (15 to 25 °C shift). We measured resting calcium levels (baseline at constant temperature, Fig. 4d) and thermal responsiveness to short-lasting 10 °C thermal up-steps (Fig. 4e). Consistent with previous studies^46,54,55^, we observed that AFD (i) produced rapid calcium transients in response to thermal up-steps, regardless of growth temperature (Fig. 4c, e) and (ii) showed higher resting calcium levels in animals grown at 15 °C compared to 25 °C (Fig. 4c, d). A 6 h shift from 15 to 25 °C did not alter the magnitude of AFD responses to short-lasting stimuli (Fig. 4c, e), but significantly increased the resting calcium level as compared to animals constantly kept at 15 or 25 °C (Fig. 4c, d). Furthermore, the differences in resting calcium levels across conditions were abolished in a calcium binding-defective version of cameleon YC2.3(D21A, D57A, D94A, D130A) (Supplementary Fig. 10e). These results highlight that resting calcium level in AFD is tonically modulated over an hour timescale and that this level is particularly high in animals in the scanning state 6 h after warming.

### Warming up-regulates *flp-6* expression in AFD to promote scanning and enhance thermotaxis on food

Since the transition to scanning after warming is linked to a tonic AFD activation and requires neurotransmission, we next wanted to identify the relevant communication molecules released by AFD. AFD produces glutamate and several neuropeptides^56,57^. We thus tested if warming-evoked scanning entry was affected in *eat-4* mutants with impaired glutamate signaling^58^ and *egl-3* mutants with impaired neuropeptide production^59^. *eat-4* mutation had no impact, but *egl-3* prevented backward frequency increase and tail bending decrease in response to 6 h warming (Fig. 4f and Supplementary Fig. 10c). These results suggest that neuropeptide not glutamate signaling is required to shift from dwelling to scanning after warming. To uncover the neuropeptide signal, we tested *flp-6* mutants. We chose this candidate because it is the most abundantly expressed neuropeptide gene in AFD^56^, its transcription is regulated by temperature and its release mediates temperature-dependent regulation on lifespan by AFD^60^. We found that *flp-6* was required to shift both backward frequency and tail bending in response to warming (Fig. 4f and Supplementary Fig. 10c). This effect was significantly rescued by a *[Pgcy-8::flp-6]* transgene selectively restoring *flp-*6 expression in AFD (Fig. 4f, Supplementary Fig. 10c). While not ruling out additional mechanisms, these data suggest that FLP-6 signal originating from AFD makes an important contribution to the warming-evoked transition to scanning.

Because FLP-6 neuropeptide is essential to modify key parameters responsible for the scanning state, which increase thermotactic performances, we then predicted that *flp-6* mutants would not perform well in locating and responding to thermogradient on food. According to our prediction, we found that, even after 1 h in a gradient, *flp-6* mutants failed to navigate to warmer side of the plate while N2 navigate within 20 min (Figs. 2f and 4j, k). When we extended the assay duration up to 3 h, we observed that *flp-6* mutants could eventually reach a biased distribution, suggesting they can encode a preferred temperature and they can produce a directed thermotaxis (Supplementary Fig. 10f). Nevertheless, the thermal preference was different than that of wild type. Therefore, whereas the altered thermotaxis performances of *flp-6* mutant could be due to an impaired ability to enter scanning, it could also be caused by an altered thermal preference encoding.

Since temperature was previously shown to affect *flp-6* transcription in AFD^60^, we next used a transcriptional reporter to test if *flp-6* transcription was actually increased in animals in the warming-evoked scanning state. We found that the fraction of animal with detectable *flp-6* reporter signal was significantly higher in animals raised at 25 °C or shifted to 25 °C as compared to animal raised at 15 °C (Fig. 4h). Furthermore, the signal intensity was significantly stronger after the 15–25 °C shift (Fig. 4g).

Collectively, our results suggest that warming-evoked transition to scanning on food is controlled by AFD and FLP-6-dependent signaling. A thermal shift from 15 to 25 °C is linked to a unique AFD state that combines elevated FLP-6 expression and chronically elevated calcium levels (Fig. 4l). Reduced calcium and low *flp-6* expression represent potential inhibitory mechanisms (red traffic lights in the model in Fig. 4l) preventing scanning entry at constant temperature. This multisite regulation only allows AFD/FLP-6-dependent scanning entry in response to recent warming on food, when this specific behavioral state is needed to jointly reach feeding and thermoregulation goals.

### Different FLP-6 receptors regulate reversal and tail bending during scanning

*C. elegans* neuropeptides function through GPCRs to generate neuromodulatory effects lasting across timescales. To identify receptors mediating FLP-6-dependent scanning entry in vivo, we examined the impact of the loss of *egl-6*, *dmsr-1* and *dmsr-7* receptor genes, coding for FLP-6 candidate receptors identified in an in vitro ligand-receptor screen^61^. We found that *egl-6* not *dmsr-1* or *dmsr-7* mutants failed to increase backward frequency, while all three mutants failed to decrease tail bending (Fig. 4I, Supplementary Fig. 10d). We further rescued defects in *egl-6* mutants by expressing EGL-6 under its native promotor. Bypassing the lack of *flp-6* by overexpressing EGL-6 under its own promoter in *flp-6* mutants was sufficient to restore their backward frequency, but not their tail bending phenotype (Fig. 4I and Supplementary Fig. 10d). Taken together, these results suggest that FLP-6 signaling might use a distributed set of receptors to orchestrate the multi-dimensional behavioral changes linked to scanning.

### FLP thermosensory neurons are essential for glocal search

**Fig. 5:**
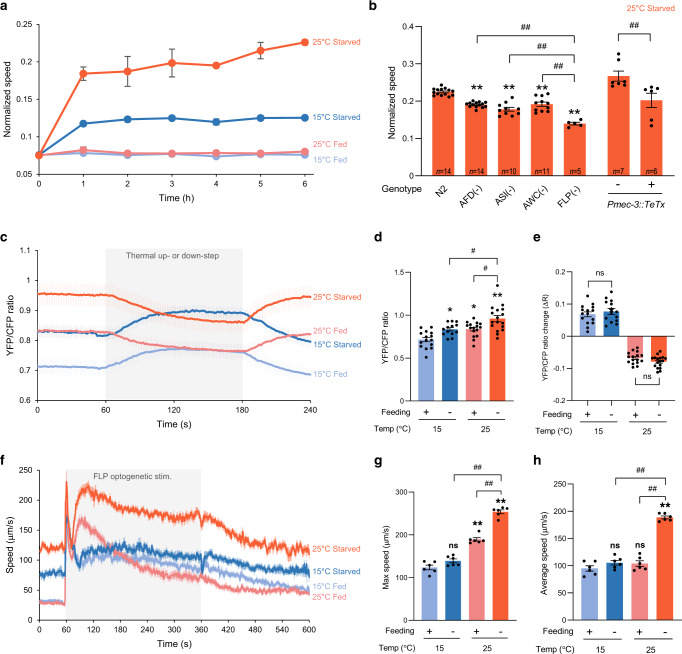
Temperature and feeding states are encoded in FLP neural pathway to control tonic speed elevation during glocal search. Speed increase time course after starvation at 15 or 25 °C and in corresponding controls left on food (**a**); mean ± s.e.m. of *n* = 8, 6, 9, 6 assays for 15 °C Fed, 15 °C Starved, 25 °C Fed and 25 °C Starved, respectively. Speed measured after 6 h of starvation at 25 °C in wild type (N2), in transgenic lines with ablated neurons, or in animals carrying a *Pmec-3::TeTx* transgene blocking neurotransmission in FLP (**b**); mean ± s.e.m. (number of independent assays, *n*, at the bottom of each bar). FLP intracellular calcium levels at rest and following 2-min 10 °C thermal up- or down-steps (**c**–**e**); mean ± s.e.m. of *n* = 15, 13, 16, 17 assays for 15 °C Fed, 15 °C Starved, 25 °C Fed and 25 °C starved, respectively (**c**), resting calcium levels (**d**), corresponding to baseline period and temperature-evoked relative calcium changes (**e**), showing FLP encodes temperature and food signals as resting calcium levels without modulating the magnitude of thermal responses to short stimuli. Impact of temperature and food on speed elevations caused by a 5-min tonic optogenetic activation of FLP (**f**–**h**). Mean speed elevation profiles (±s.e.m. of *n* = 6 assays, each scoring *≥*30 worms) with a first spike corresponding to an initial high reversal period (*t* = 60-90 s) and a second long-lasting speed elevation period (90-360 s) (**f**). Max (**g**) and average speed (**h**) during the long-lasting speed elevation period. *p < 0.05 and **p < 0.01 versus 15 °C Fed condition; #*p* < 0.05 and ##, *p* < 0.01 versus the indicated condition by Bonferroni posthoc tests (**b**, **d**, **e**, **g**, **h**). ns not significant. Source data are provided as a Source Data file.

Our systematic characterization of food and temperature-dependent behavioral states (Figs. 1–3) showed that temperature strongly impacts the choice between global and glocal search. Since speed and omega turn up-regulation are distinctive characteristics of glocal search (Fig. 5a, Supplementary Fig. 6 and 11a) and suffice to recapitulate the main navigational features of this exploration strategy in simulations (Supplementary Fig. 8), we focus on these two parameters for further analyses.

To identify neurons required for the transition to glocal search at 25 °C, we genetically ablated candidate thermosensory neurons (Fig. 5b and Supplementary Fig. 11b). Ablation of FLP neurons caused severe, while ASI ablation caused moderate impairment of both speed and omega turns. AFD and AWC ablation caused moderate impairment of only speed not omega turn frequency. Blocking synaptic transmission with Tetanus toxin expression in FLP also prevented the elevation of both speed and omega turn frequency (Fig. 5b and Supplementary Fig. 11b). These results highlight a major contribution of FLP neurons in up-regulating speed and omega turns for transition to glocal search.

### Food and temperature affect FLP resting calcium level and downstream circuit responsiveness

FLPs are non-adapting phasic and tonic thermosensory neurons^62^. To examine if FLP neurons could jointly encode food and thermal information, we performed in vivo calcium imaging of FLP using the ratiometric YC2.3 sensor. Consistent with our previous work^62^, we found that FLP encodes information of absolute temperature (15 °C and 25 °C) as resting baseline calcium levels (Fig. 5c, d). Starvation also increased resting calcium in FLP, having an additive effect with temperature. Maximal levels were thus observed in animals in glocal search state after starvation at 25˚C. Like for AFD, the differences in resting calcium levels across conditions were abolished in a calcium binding defective version of the sensor (Supplementary Fig. 11c). In contrast to the resting calcium levels, we found that responses of FLP to 2-min, 10 °C up- or down-steps were neither affected by starvation, nor by temperature (Fig. 5c and e). These results indicate that starvation and temperature independently modulate resting calcium levels (not thermal responsiveness) of FLP neurons, which could partially encode information of distinct behavioral states.

To examine if the responsiveness of components downstream of FLP depolarization are modulated in specific states, we monitored the changes in speed evoked by the optogenetic activation of FLP as a function of temperature and feeding state. Consistent with our previous work (performed in animals starved at 23 °C^62^), we found that optogenetic FLP activation first triggered an abrupt speed change associated with a high reversal phase (60–90 s), which was followed by a prolonged speed induction phase (90–360 s) persisting throughout the stimulation (Fig. 5f). Both fed and starved animals at 15 °C produced a relatively low speed elevation with a slow decay after reaching max speed (Fig. 5f and g). Fed animals at 25 °C produced a more pronounced increase compared to animals at 15 °C (Fig. 5f and g), but this speed elevation in the steady phase underwent fast decay (resulting in low average speed measures Fig. 5h). Starved animals at 25 °C showed further increase in maximum speed compared to animals on food with minimal decay (Fig. 5f and g), resulting in elevated average speed (Fig. 5h).

Collectively, results from calcium imaging and optogenetic analyses suggest a model in which the temperature and starvation-dependent sustained speed increase underlying glocal search could result from a concomitant increase in FLP tonic activity and enhanced responsiveness of downstream components. In corollary, the lower speed observed in dwelling and global search states seems to result from the inhibition of the FLP-dependent speed-promoting pathway by food and low temperature.

### Dopamine prevents search behavior on food at high temperature

**Fig. 6:**
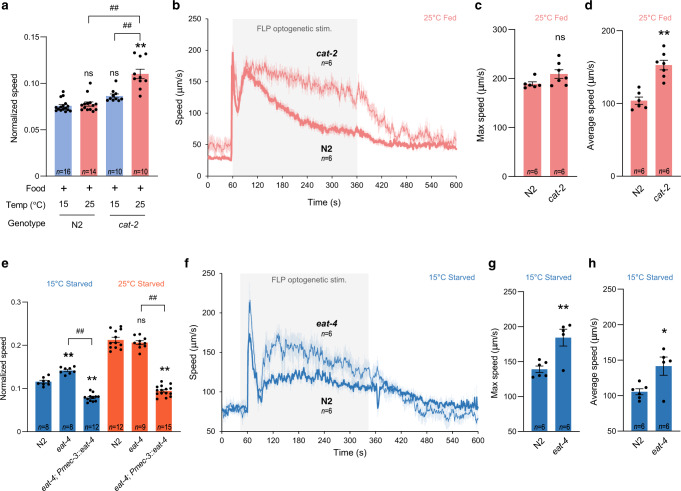
Speed elevation is inhibited by dopamine in fed animals at 25 °C and by glutamate signaling in starved animals at 15 °C. Impact of a *cat-*2 mutation blocking dopamine biosynthesis (**a**–**d**) on the speed of fed animals at 15 or 25 °C (**a**), and on the long-lasting speed response evoked by a 5-min optogenetic activation of FLP in animal maintained at 25 °C (**b**–**d** scored and reported as in Fig. 5). Similar analyses for an *eat-4* mutation affecting glutamatergic signaling in starved animals at 15 °C (blue) or 25 °C (orange) (**e**–**h**). Data as mean ± s.e.m.; indicated *n* correspond to independent assays, each scoring ≥30 animals (**a**–**h**) *p < 0.05 and **p < 0.01 versus 15 °C Fed condition or respective N2 control; #*p* < 0.05 and ##, *p* < 0.01 versus the indicated condition by Bonferroni posthoc tests. ns not significant. Source data are provided as a Source Data file.

Dopamine signaling was shown to modulate food-dependent behaviors^21,23,34^. Next, we tested if dopamine signaling inhibits temperature-dependent behaviors on food. Unlike wild type, *cat-2* mutants defective for dopamine synthesis had increased speed and omega turn frequency at 25 °C compared to 15 °C even on food (Fig. 6a and Supplementary Fig. 12a). Moreover, the fast speed decay observed during FLP optogenetic stimulation at 25 °C on food was also revoked in *cat-2* mutants (Fig. 6b–d). These results suggest that active dopamine signaling on food normally inhibits high temperature-evoked behaviors and confirms that it functions as an important regulatory step in the context of the transition from dwelling to search behaviors.

### Glutamate signal from FLP hinders speed increase during global search at 15 °C

Next, we hypothesized that one or more signals from FLP could modulate speed and omega turns after starvation, keeping in mind that such signals could either act by inhibiting the response at 15 °C or stimulating it at 25 °C. Since FLP produces glutamate, we first examine starved *eat-4* mutants and found they displayed increased speed at 15 °C (Fig. 6e). A *[Pmec-3::eat-4]* transgene restoring *eat-4* expression in FLP neurons could revert the speed to values that were even below those in wild type, potentially due to *eat-4* over-expression (Fig. 6e). The *eat-4* mutation also potentiated the long-lasting speed elevation during optogenetic stimulation of FLP neurons, with an increase in maximum and average speed at 15 °C (Fig. 6f–h). In fed animals the loss of *eat-4* produced no effect on speed and omega turns (Supplementary Fig. 13). These results suggest that glutamate signaling in the FLP-dependent neuronal pathway functions as temperature-dependent inhibitory signal for speed after starvation and might be relevant to prevent maximal speed elevation at 15 °C, staying at an appropriate level for global search.

### FLP-5 and other neuropeptides from FLP promotes glocal search

**Fig. 7:**
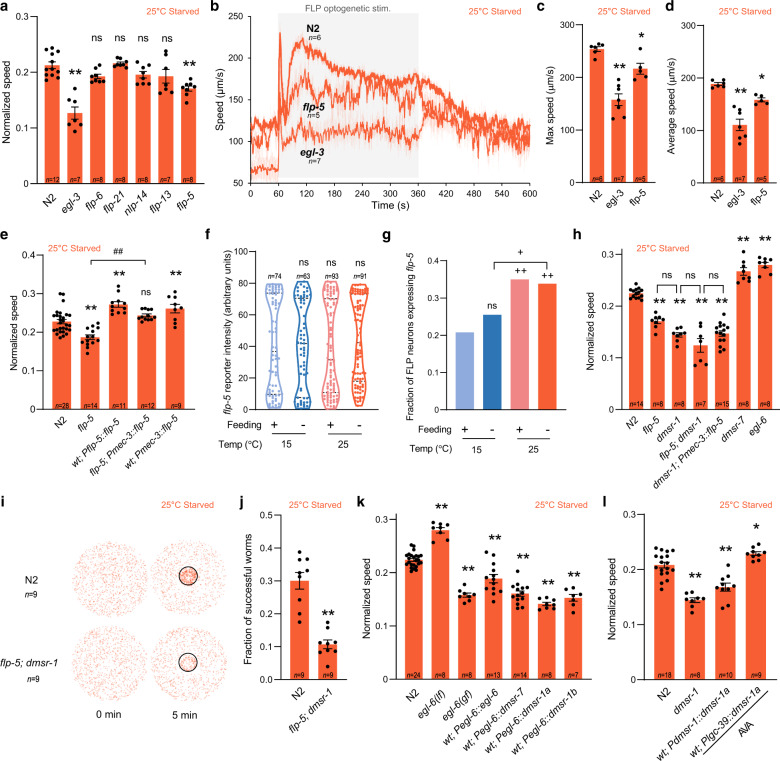
FLP-5/DMSR-1 signaling mediates speed elevation to promote glocal search. Impact of neuropeptide-affecting mutations on the speed of starved animals at 25 °C (**a**) and after a tonic optogenetic FLP stimulation reported as in Fig. 5 (**b**, **c**, **d**). Impact of *flp-5* mutation, over-expression, and rescue/over-expression with *Pmec-3::flp-5* transgene expressed in FLP (**e**) and of loss-of-function mutations affecting FLP-5 receptors (**h**) on the speed of starved animals at 25 °C. *flp-5* transcriptional reporter quantification comparing mean intensity (**f**) and fraction of FLP neurons with detectable signal (**g**). Food drop assays reported as in Fig. 2 showing reduced food-reaching performances in *flp-5; dmsr-1* (**i**, **j**). Impact of gain-of-function (gf) and loss-of-function (lf) mutations in *egl-6*, as well as FLP-5 receptor over-expression in *egl-6-*expressing cells, revealing that EGL-6, DMSR-1a, DMSR-1b and DMSR-7 have a similar inhibitory effect on the speed of starved animals at 25 °C (**k**). Opposite impact of broad DMSR-1 overexpression in *dmsr-1-*expressing cells or AVA-specific overexpression, respectively, on the speed of starved animals at 25 °C (**l**). Data as average ± s.e.m. with number of independent assays, *n*, indicated on each graph (**a**–**e**, **h**, **j**, **k**, **l**). *p < 0.05 and **p < 0.01 versus 15 °C Fed condition or respective N2 control; #*p* < 0.05 and ##, *p* < 0.01 versus the indicated condition by Bonferroni posthoc tests. +*p* < 0.05 and ++*p* < 0.01 versus 15 °C Fed or indicated condition by Fisher’s exact test for contingency comparisons. ns not significant. Source data are provided as a Source Data file.

Next, we analyzed the contribution of neuropeptide-based signaling. *egl-3* mutation prevented the increase in speed after starvation at both 15 and 25 °C (Fig. 7a, and Supplementary Fig. 13a) and in omega turn frequency (Supplementary Fig. 13b and 14a) after starvation at 25 °C, indicating requirement of neuropeptide signaling in controlling global and glocal search. We focused on the regulation of glocal search and performed a biased screen for neuropeptide-encoding genes expressed in FLP neurons. We found that the up-regulation of speed upon starvation at 25 °C required *flp-5*, while that of omega turns required *flp-5* and *nlp-14* (Fig. 7a and Supplementary Fig. 14a). Both *egl-3* and *flp-5* mutations significantly impaired the long-lasting speed elevation evoked by FLP optogenetic stimulation (Fig. 7b–d). Both speed and omega turn defects were rescued by a *[Pmec-3::flp-5]* transgene, driving *flp-5* expression in FLP (Fig. 7e and Supplementary Fig. 14b). Moreover, overexpression of *flp-5* under its native promoter or in FLP using the *mec-3* promoter caused further increase in speed and omega turn frequency compared to wild type (Fig. 7e, and Supplementary Fig. 14b). While our data suggest several neuropeptides play a role and do not rule out additional sources for FLP-5, they indicate that FLP-originating FLP-5 represents a relevant signal promoting the transition to glocal search.

### *flp-5* transcription is controlled by temperature in FLP

Since FLP-5 is a speed-promoting signal from FLP, we next asked if *flp-5* transcription depends on temperature and/or feeding status. When imaging a *[Pflp-5::mNeonGreen]* transcriptional reporter in FLP, we observed a bimodal distribution of signal intensity among cells with detectable expression that was similar in all conditions (Fig. 7f), but the fraction of animals with detectable *flp-5* reporter expression was significantly higher at 25 °C compared to 15 °C regardless of feeding status (Fig. 7g). Considering that *flp-5* overexpression with *mec-3* promoter was sufficient to up-regulate speed and omega turns (Fig. 7e and Supplementary Fig. 14b), we propose that reduced *flp-5* transcription in FLP at 15 °C may be one of the inhibitory mechanisms preventing the transition to glocal search at this temperature.

### FLP-5/DMSR-1 signaling is essential for glocal search

In vitro ligand screen data indicate that FLP-5 is a potent ligand of EGL-6 and DMSR-7 and activates DMSR-1 with lower potency^61^. To identify functional FLP-5 receptors in vivo, we tested *egl-6*, *dmsr-7* and *dmsr-1* mutants. Speed and omega turn frequency was increased in *egl-6* and *dmsr-7* loss-of function mutants, which is opposite to the effect in *flp-5* mutant (Fig. 7h and Supplementary Fig. 14c), suggesting that EGL-6 and DMSR-7 are relevant for these phenotypes, but are unlikely to directly mediate the glocal search-promoting effects of FLP-5. In contrast, *dmsr-1* loss-of-function mutants displayed decreased speed and omega turns frequency. *flp-5; dmsr-1* double mutants did not significantly differ from *dmsr-1* single mutants. Additionally, the speed and omega turn frequency increase caused by *flp-5* over-expression in FLP was blocked in a *dmsr-1* mutant background (Fig. 7h and Supplementary Fig. 14c). We also confirmed the importance of FLP-5/DMSR-1 signaling for glocal search with our food drop assay, and showed that, as expected based on the marked impact on both speed and omega turn, the double *flp-5;dmsr-1* mutants performed very poorly (Fig. 7i, j). Taken together, these results are compatible with a model in which FLP-5 neuropeptide functions through DMSR-1 receptor to shift speed and omega turn frequency in order to support an efficient glocal search strategy.

### DMSR-1 and -7 function as inhibitory receptors in vivo

Next, we wanted to address if FLP-5 receptors work as excitatory or inhibitory receptors. EGL-6 is a well-established inhibitory receptor^63^ and recent studies suggests an inhibitory role for DMSR-7^64^ and DMSR-1^65^ as well. However, the two isoforms encoded by *dmsr-1* vary at their C-termini and could potentially activate different G-protein subtypes, calling for further analyses. We designed experiments to compare the effect of DMSR-1a/b and DMSR-7 to that of EGL-6. *egl-6(n4537)* loss-of-function mutation increased, whereas *egl-6(n592)* gain-of-function mutation decreased speed and omega turn frequency after starvation at 25 °C (Fig. 7k, Supplementary Fig. 14d). Overexpression of *egl-6* under its own promoter was also sufficient to down-regulate speed and omega turns. These results indicate that overexpressing an inhibitory receptor in *egl-6*-expressing cells negatively impacts speed and omega turn, hence providing a way to test if other receptors have the same inhibitory effect. The over-expression of DMSR-1a, DMSR-1b, as well as DMSR-7 all produced an inhibitory impact similar to EGL-6 over-expression (Fig. 7k and Supplementary Fig. 14d). Therefore, the FLP-5 receptors identified in vitro (EGL-6, DMSR-1 and DMSR-7) display inhibitory activity in vivo when over-expressed in the circuit relevant for speed control.

### DMSR-1 activity in AVA command interneuron promotes speed elevation

**Fig. 8:**
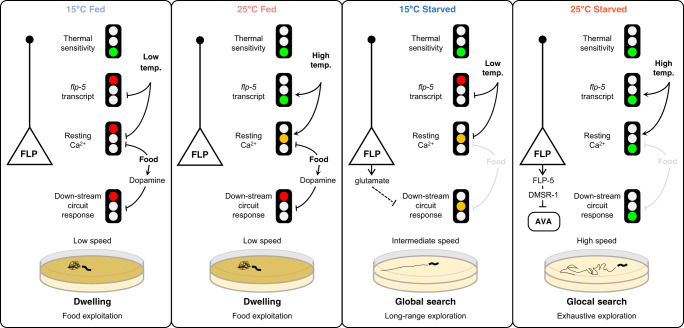
Multi-site regulation in the FLP pathway controls speed elevation during glocal search. Model for food and temperature-dependent control of exploitation/exploration strategies via multi-site gating mechanisms (traffic lights). On food, animals dwell regardless of temperature with one or more gates closed in the FLP-pathway (red lights). During global search after starvation at 15 °C, only parts of the gates are lifted (green lights), still preventing the up-regulation of speed, which is also hindered by FLP glutamate signaling. After starvation at 25 °C, all gates are lifted and FLP tonically signals via FLP-5/DMSR-1 to elevate speed and promote glocal search.

Our next goal was to determine the locus of action of DMSR-1 receptor in controlling the speed elevation during glocal search. DMSR-1 is broadly expressed in the nervous system^56^, including in forward locomotion-promoting neurons (like AVB, RIB and DVA), as well as backward locomotion-promoting neurons (like AVA, whose activation was also shown to reduce animal speed^66^). Based on these observations, we made two predictions. First, since DMSR-1 is expressed in neurons with antagonistic impact on speed, then the FLP-5 signal relevant for the speed increase is unlikely to have a broad impact that generally affects all DMSR-1-expressing neurons. Consistent with this view, overexpressing DMSR-1a or b with its endogenous promoter failed to up-regulate speed and actually reduced it, suggesting that FLP-5 must act via a narrower subset of target cells in order to elevate speed (Fig. 7l). Second, since FLP-5 peptides activate DMSR-1 with an EC50 value in the micromolar range and DMSR-1 is inhibitory, its relevant place of action downstream of FLP-5 is likely to be speed-inhibiting neurons that are post-synaptic to FLP. AVA command interneuron was our top candidate and we found that AVA-specific overexpression of DMSR-1 primarily in AVA with the *lgc-39* promoter was indeed sufficient to increase speed (Fig. 7l). Taken together, these results elucidate an important signaling mechanism participating in the orchestration of the behavioral transition to glocal search, which involves the FLP-5/DMSR-1-dependent regulation of AVA by FLP (Fig. 8).

## Discussion

The ability to engage in persistent behavioral states and to switch between different states in response to external and internal multimodal cues is an essential animal capability to improve growth and survival via the execution of context-dependent behavioral strategies. Owing to the complexity of the signaling involved and of the behavioral responses themselves, we still know very little about the neural and molecular mechanisms that orchestrate coherent and long-lasting multidimensional changes, which permit to implement these behavioral strategies. Here, we systematically decode how food availability is integrated with past and recent thermosensory experience and show how these factors interact, leading the animal to select between different strategies. We confirmed previously described persistent behavioral states (dwelling and global search)^16,17 ^highlighted novel exploitation and exploration states (scanning and glocal search, respectively) and illustrate their potential ecological benefits. Our dissection of the neural and molecular pathways involved provides two separate examples in which food and/or temperature signals converge to release inhibitions at multiple regulatory sites. This allows for tonic neuropeptide signaling by specific sensory neurons (AFD and FLP, respectively) to be only engaged when several conditions are met. The system dynamics might be tuned to cause animals to ignore short-lasting stimuli when committing to persistent behavioral states. But quite remarkably, the system still allows for a parallel control of short-term behavioral responses via phasic signaling by these same sensory neurons. The similarities between the two examples in AFD and FLP, respectively, suggest a potentially general regulatory logic through which sensory pathways integrate multimodal context to control behavioral responses over different timescales.

### Multimodal context-dependent exploitation/exploration strategies

Like in previous studies^67,68^, our results suggest that behavioral states under different contexts can be represented as a unique code of *C. elegans* locomotion and posture. On food, worms are in a dwelling state^15,16,19^, regardless of different long-term growth temperature. Over a shorter timeframe, however, worms transiently enhanced dwelling state in response to cooling, while they persistently transition to a scanning state in response to warming. The asymmetry in the nature and duration of the responses to cooling and warming, respectively, reveals differential prioritization of environmental cues and specific behavioral strategies affecting the exploitation-exploration trade-off when adapting to a changing environment.

The scanning state during which animals produce more frequent bouts of backward locomotion is different from the roaming behavior in which animals move faster and down-regulate reversals^14^. Whereas transition to scanning is less drastic than the transition to off-food search modes, animals in the scanning state sample their local environment more exhaustively than dwelling animals and, when confronted to a thermal gradient, produce more efficient thermotaxis. We speculate that short motion bouts during scanning may improve gradient detection, because motion-evoked thermal changes are the primary inputs through which worms decode their thermal environment^69^. In previous studies, animal fitness was reduced when exposed to cycling temperature regimes, demonstrating that physiological adaption to new temperatures is not costless^48^. With little energy invested in locomotion and without disrupting local food exploitation, the scanning state could potentially facilitate the detection of thermal gradients and help reach previous growth temperature in a slow-developing thermotactic drift that will limit the need for long-term physiological adaptations.

After prolonged starvation, current temperature influenced the persistent transition to the previously known global search state at 15 or to the presently described glocal search state at 25 °C. During global search, animals increase forward movement at an intermediate speed and suppress turns, resulting in a long-range navigation with only shallowly curved trajectories to locate food. During glocal search, concerted up-regulation of speed and omega turns results in a specific exploration strategy, where animals exhaustively sample local environment by making frequent turns while still performing long-range navigation due to marked speed increase. Glocal search enables finding food faster than global search, but is energetically expensive. We speculate that worm physiology and metabolism at 25 °C puts worms under a higher time pressure, justifying the allocation of more resources for exploration.

The definition of well-separated behavioral states made in the present study, likely results from the choice of strongly contrasting conditions (15 vs 25 °C and presence vs absence of food). Future studies using more granular or smoother thermal changes, as well as partial food deprivation over different timescales may contribute to define if behavioral states develop on a continuum like previously suggested^68^. Nevertheless, we believe that the definition of discrete behavioral states is conceptually important, as they permit the discussion of specific ecological advantages associated with specific behavioral patterns.

As a whole, our study (i) expands the repertoire of described exploitation/exploration strategies in *C. elegans*, (ii) confirms the importance and efficacy of specific strategies, (iii) emphasizes the need for context-dependent transition between them, and (iv) expands our understanding of their multidimensional nature by comprehensively characterizing their underlying behavioral codes.

### Multisite regulation in tonic sensory pathways integrates multimodal context

Although our data also suggest that additional pathways are involved, it is quite remarkable that the food and thermal contexts are largely encoded within AFD and FLP-dependent pathways. These mediate the transitions to scanning and to glocal search, respectively, and control both fast- and progressively-developing responses. Tonic signaling in these pathways, via specific neuropeptides, seems to have an instructive role and to be sufficient to trigger changes in many behavioral parameters. It is therefore important that robust inhibitory mechanisms prevent their action under conditions where these strategies should not be engaged.

The transition to scanning requires AFD, which signals via FLP-6 neuropeptide. In scanning animals, AFD thermal responsiveness to short thermal stimuli is not affected, but AFD is in a particular state combining (i) sustained tonic activity (with high resting calcium levels) and (ii) increased *flp-6* expression. During dwelling at constant temperature, neither increased AFD resting calcium (at 15 °C), nor increased *flp-6* transcript levels (at 25 °C) seem sufficient to trigger scanning entry. Only after a recent warming, does AFD show synergistic increases in steady state calcium and *flp-6* transcript levels (green lights in Fig. 4l and Supplementary Fig. 15). Whereas our data do not rule out additional modulation mechanisms downstream of AFD, they are consistent with a model in which multiple intracellular gates located at the sensory neuron level need to be lifted in order for the animal to shift its behavioral state (Fig. 4l). This regulatory mechanism adds to the complexity of the cell-autonomous thermal history computing capability of AFD neurons, which have been revealed in multiple previous studies^55,70–73^.

The transition to glocal search is promoted by FLP, via FLP-5 and most likely also via additional unidentified neuropeptides. The FLP pathway integrates temperature and food signals to promote the transition to glocal search only at high temperature and in the absence of food. Like for AFD, the FLP thermal responsiveness to short stimuli was not different between behavioral states, indicating that the distinction between states is encoded downstream of the FLP thermo-detection process. Glocal search was associated with (i) a very high resting calcium level in FLP, (ii) elevated *flp-5* transcription and (iii) increased responsiveness of the circuit downstream of FLP activation (Fig. 8). Given the likely instructive role that tonic FLP-5 signaling from FLP has in the transition to glocal search, each of these three processes might represent a potential regulatory gate able to prevent this transition (Fig. 8 and Supplementary Fig. 15). Indeed, in dwelling animals at 15 °C on food, all three gates are closed with low baseline FLP calcium, low *flp-5* transcript and low responsiveness of components downstream of FLP. In animals performing global search at 15 °C off-food, we only observed a partial increase in FLP steady-state calcium levels, while the rest of the gates were unchanged compared to dwelling animals on food at 15 °C. Furthermore, glutamate signaling from FLP contributes to prevent speed increase. Several neurons (ASI, ASK, AIA, AVK)^17,34,53^ have been implicated in transition to global search and probably FLP signals information of low temperature in this context. In dwelling animals at 25˚C on food, we observed a partial increase in FLP resting calcium levels and an increase in *flp-5* transcript, but dopamine signaling reduced the responsiveness of components downstream of FLP, which might be the most important gate in this context in order to block the high temperature signals. Ultimately, the glocal search mode is associated with a unique state in the FLP pathway where food absence and current temperature signals converge to lift all the cellular gates in order to produce context-specific persistent behavioral transitions (Fig. 8). Of note, with the term “gate”, we don’t intend to mean here that each regulatory step is a ON/OFF switch, but simply that an inhibition at every step may suffice to strongly down-regulate the signaling pathway outcome.

Taken together, the two examples of AFD- and FLP-dependent behavioral transitions described here, suggest a generally applicable regulatory logic, where multisite regulatory gates, qualitatively and quantitatively affecting tonic signaling and its reading in sensory circuits, function to ensure temporal and multimodal integration of signals. The system will thus enable behavioral transitions to occur only in response to coherent and sustained changes in the environment.

### A distributed network of inhibitory GPCR(s) orchestrates behavioral transitions

The progressive increase in complexity throughout our study limited our ability to dissect all the downstream molecular and cellular components of the entire behavioral code that is modulated during different behavioral state transitions. However, we could identify functional receptors for FLP-6 and FLP-5 as well as one relevant cellular locus of action for the latter, hence providing a partial, yet suggestive picture of the downstream effector functional logic.

Tonic FLP-6 signaling from AFD was previously shown to mediate the temperature effect on lifespan via unknow receptors^60^. Here we show that FLP-6 might promote the transition to scanning, through at least three inhibitory GPCRs broadly expressed in the nervous system: EGL-6, known to regulate egg-laying^63^, DMSR-1, known to regulate stress-induced sleep^65^, and DMSR-7, recently proposed to control sickness behavior^64^. Our data suggest that FLP-6 mediates a progressive increase in backward frequency through EGL-6 and a fast decrease in tail bending through DMSR-7, DMSR-1, and EGL-6. Therefore, different FLP-6 receptors contribute to regulate different behavioral parameters over different timescales to ensure transition and maintenance of the scanning state.

Our study also sheds light on some downstream mediators of the FLP pathways. During transition to glocal search, FLP-5 might function through DMSR-1 to inhibit AVA and unidentified neurons to increase speed and omega turns, respectively. Interestingly, speed up-regulation entails the inhibition of an inhibitory pathway, suggesting that the animal default state under favorable conditions would be a metabolically inexpensive low arousal state. Moreover, fine-tuning the neural circuit activity by modulating the inhibition of the key command interneuron AVA^30,74^ can enable the transition to a metabolically expensive but adaptive persistent behavioral state.

In conclusion, our study suggests that *C. elegans* continuously weights the sensory valence of temperature in distinct thermosensory neurons, based on past experience, as well as internal and external contexts. In the thermosensory circuit, cellular and molecular multi-site regulatory gates act in concert to generate physiologically adaptive behavioral states and balance exploration-exploitation strategies. Moreover, only in specific contexts does the GPCR(s) dependent fine-tuning of excitation-inhibition balance mediate persistent behavioral state transitions. Given the prevalence of tonic sensory signaling, the widespread expression of GPCRs and their emerging functions in brain state pathologies (i.e., anxiety, depression, schizophrenia)^75–77^, we propose that similar multi-site regulatory gating-dependent integration mechanisms converging on GPCRs might mediate persistent behavioral state transitions in response to a variety of internal and external cues and in additional species.

## Methods

### *C. elegans* growth, maintenance and synchronization

*C. elegans* strains were maintained according to standard techniques on nematode growth medium (NGM) agar plates seeded with OP50. Animal synchronization was made by treating gravid adult with standard hypochlorite-based procedure. Strain List section below includes a list of strains used in the present study.

### Behavior analyses

An overview of the device and pipeline for behavioral recording and analyses is presented in Supplementary Fig. 1a and b, and detailed below.

### Behavioral recordings

*Video recordings-* Behavior of animal populations crawling on solid medium plates was recorded in a custom-made temperature, vibration and illumination-controlled platform (Supplementary Fig. 1a). High-resolution (2448×2048 pixels), 3-min movies (~50 animals/movie) were recorded using DMK33UX250 camera (The Imaging Source), at 10 frames per second as a snapshot of the behavioral state in given condition using IC Capture software (The Imaging Source) and saved as *.avi* files. All the behavioral recordings across conditions and genotypes were performed in young adult animals (~800 µm in length). Each condition and genotype were recorded on at least 3 separate days with multiple replicates each day. All the behavioral experiments were performed in standard 6-cm NGM petri dishes (with or without *E. coli* food lawn) in a lid-on setting unless otherwise mentioned.

*Steady-state behavior-* Animals were grown and recorded without changing the temperature throughout life for steady-state/baseline behavior at 15 °C or 25 °C, respectively. For detailed behavioral state characterization young-adult wild type animals were recorded at 15 °C from 93–99 h, and at 25 °C from 49–55 h for 6 h. For mutants, steady state baseline behavior was recorded once in young adult animals.

*Temperature shift experiments-* For temperature shift experiments, animals were moved to the recording device pre-set at desired recording temperature just before the recording. Desired recording temperature of 15 °C or 25 °C on shifted recording plates was achieved within 45 min after temperature shift. Duration of transition was counted from the time of plate shift. Wild-type animals for detailed behavioral state characterization were recorded every hour for 6 h. Mutants were only recorded after 6 h of temperature shift to focus on steady behavioral states.

*Starvation experiments-* Fed animals were washed 3 times in 1.5 mL collection tubes with distilled water (pre-equilibrated at growth temperature of worms) to remove OP50. During washes worms were left to settle to the bottom of the tubes by gravity. About 50–80 animals were then plated on NGM plates with a drop of water and left to airdry for 5 min. Total duration of wash and drying was ~10 min per plate. Duration of starvation was considered from the time of start of the wash. Wild-type animals for detailed behavioral state characterization were recorded every hour for 6 h. Mutants were only recorded after 6 h of starvation to focus on steady behavioral states.

*Preparing transgenic animals for behavioral recordings-* Transgenic animals were picked 24 h before recordings and placed to equilibrate in the recording device.

### Behavioral parameter extraction from movies

Recorded movie files were analysed using the Tierpsy tracker v1.4 for detailed quantification of locomotion and posture^78^. The output feature file contains average value for ~700 behavioral parameters for all tracked worms. Previous work recommended using a smaller parameter subset with 256 Tierpsy features for balancing power and interpretability^27^. We reasoned that this list of 256 parameters is suitable for the studies where high-power is required to capture all the possible phenotypic difference (e.g. genetic or drug screens). However, this total number of is still relatively large to handle in a study performing multiple subsequent mechanistic dissections and still contains many parameters which are either (i) difficult to interpret, (ii) inherently noisy, or (iii) still redundant with others. We used an alternative approach to decrease the list of Tierpsy v1.4 parameters (~700) using the following reasoning:

1. Include all the parameters accounting for major postural and locomotion variations that are likely to have a neural basis and an interpretable ecological impact (bending of posture at different body parts, eigen projections, speed, omega turns, reversals, forward and pause locomotion)
2. Remove redundancy wherever possible (only used abs value and excluded positive and negative value of the same parameter)
3. Remove noisy parameters with empty values for most datapoints (as upsilon turn frequency).
4. Keep sufficient parameters to grasp complete information about a behavioral cluster (backward frequency, backward time, inter backward time, backward time ratio)
5. Further remove redundant parameters of a behavioral cluster that are likely to have the same mechanistic basis (removed backward distance, inter backward distance, backward distance ratio based on the logic that backward time and distance parameters might have same underlying mechanism)
6. Only keep information of a behavioral parameter for the individual body parts where required (e.g., all the bending parameters kept for individual body part), else only information of midbody is used (e.g., only keep midbody speed but not tail speed).
7. Length normalized speed was introduced later in the study to control for growth/age dependent variations in speed across conditions and genotypes.

Applying the reasoning, we focused on a set of 47 (17 postural, and 30 motion, as described in Supplementary Fig. 1c) readily interpretable behavioral parameters, which we reasoned best explain all the changes, likely to have a directly addressable mechanistic basis and can represent behavioral states of the animals.

### PCA and hierarchical clustering

We performed a global Principal component analysis (PCA) on raw average values of 47 behavioral parameters examined across all the wild type conditions in our study (Figs. 1, 3, Supplementary Fig. 2, Supplementary Data 3–5). In a secondary set of analyses, we repeated two PCA analyses: one for a subset of 30 motion parameters (Supplementary Fig. 3) and second on a subset of 17 postural parameters (Supplementary Fig. 4) as defined in Supplementary Fig. 1. Phenotypic clustering (Fig. 3, Supplementary Fig. 5) was performed on *z*-score normalized values of each parameter across all conditions, for all the behavioral parameters. PCA and hierarchical clustering was performed using Clustvis webtool by default SVDimpute algorithm^79^. This quantitative comparison across all the conditions allowed us to map behavioral states in a common multidimensional space.

### Optogenetics

Optogenetics experiments in temperature-controlled environment, were performed using a custom-made plug-in device attached to our high-content behavioral recording platform (Supplementary Fig. 1a). Homogeneous blue light stimuli were delivered though a ring of blue LED (460 ± 10 nm). Power was adjusted with an optocoupler-based system and the delivered light intensity (15 W/m^2^) was determined with a portable spectrophotometer (USB4000, OceanOptics). Animals were grown on all-trans-retinal (ATR)-containing plates. 0.1% (v/v) of ATR stock (100 mM, in ethanol) were added to an OP50 bacteria suspension and 6-cm NGM agar plates were each seeded with 250 µl of this mix^80^. We previously published control experiments in the absence of ATR showing no behavioral response at this light intensity^62^. Animals were recorded for 600 s (60 s baseline, 300 s light-stimulation, 240 s recovery). Maximum and average speed elevation were calculated only during for the time span between *t* = 90 and 360 s in order to exclude the first phase of reversal bursts observed at the onset of FLP stimulation. The speed data for optogenetic experiments were obtained as Tierpsy timeseries feature output ‘abs midbody speed’ and represents absolute average speed (in forward or backward locomotion) of all the animals at each timepoint.

### Locomotion trajectory analysis and simulation

Real locomotion trajectories (1 min) were calculated based on *x*–*y* coordinate data of 35 randomly sampled animals in each behavioral state. Trajectory Monte–Carlo simulations were performed with a custom-made excel sheet derived from a previous work^44^ and available as Supplementary Data 2. A random walk in an isothermal environment was simulated, where animals will make stochastic reorientation events and, in between, move in straight lines at a defined constant speed. Simulation inputs were the animal speed, the frequency of omega turns and the frequency of reversals. Input values for each condition/behavioural state were taken as the average empirically measured values. Distance represents summed path travelled and displacement represents the length of a linear vector connecting the starting and the end points of a 1-min trajectory.

### On-food thermotaxis assay

The on-food thermotaxis assay is a modified version of previously described thermotaxis assays on linear gradient usually carried out on food-free plates, with freshly food-deprived animals and lasting <1 h^50^. Here, experiments were performed on food, without worm transfer procedure and lasted longer (up to 3 h). Animals were transferred to experimental NGM plates completely and evenly covered with OP50 24 h before recordings. Plates maintained at mentioned isothermal conditions were gently transferred to a stable linear thermal gradient (0.5˚C/cm) created on an aluminium plate with a custom-made thermalized system. This system included Peltier elements controlled by a proportional integrative derivative (PID) controller (McShane Inc., USA)^44^. Following the worm plate transfer, the thermal gradient was established within 10 min. Movement was captured once before and at multiple timepoints after transfer using DMK 21BU04 camara (The imaging source). The entire experiment was performed in a lid-off setting to monitor temperature in the gradient using an infrared thermometer. Room humidity ranged from 40 to 50%. Coordinate data of worms were obtained by manually spotting in each image using ImageJ. On-food thermotaxis index was calculated as the mean of the population distribution normalized to the plate size (values ranging from 0 to 1). An index value of 0 would correspond to an extreme cryophilic bias with all the worms at the cool end of the plate; a value of 1 would correspond to an extreme thermophilic bias with all the worms at the warm end of the plate; a value, 0.5 corresponds to an even distribution on both side of the plate center (like at the start of each experiment).

### Food drop assay

Animals were starved at 15 °C or 25 °C on 6-cm NGM plates as described above. After 6 h of starvation (at 15 °C or 25 °C) a dense 20 µL drop of OP50 (OD600 after 1:100 dilution: 0.57 A) was dropped at the center of the plate. Experiments were performed in lid-on settings and lid was only removed once to add the OP50 drop. Snapshots of worm distribution were taken before and after at desired timepoints. Coordinate data of worms was obtained by manually spotting worms in each image using ImageJ. Region of success was defined as a circular region of 1.6 cm diameter around the food drop. Animals already present at the region of success before dropping the food were discarded from the analysis. Fraction of successful food search was calculated using the following formula:

Fraction of successful food search = Worms in region of success/Total number of worms

### Calcium imaging

Calcium imaging experiments in AFD and FLP were performed in a temperature-controlled CherryTemp microfluidic system (Cherry Biotech) and analysed as previously described^54,62^. Briefly, adult worms were glued and imaged using a Leica DMI6000B inverted epifluorescence microscope equipped with a HCX PL Fluotar L40x/0.60 CORR dry objective, a Leica DFC360FX CCD camera, an EL6000 light source, and equipped with fast filter wheels for FRET imaging (excitation filter: 427 nm (BP 427/10); emission filters 472 (BP 472/30) and 542 nm (BP 542/27)). YFP/CFP ratio of the YC2.3 cameleon indicator was used to compare resting calcium levels across conditions, as well as relative changes in response to short-lasting stimuli. When the impact of starvation and thermal shift were examined, recordings were made 6 h after the condition changes. A calcium binding-defective version of YC2.3 was created by gene synthesis to introduce the following four mutations affecting each of the four EF-hand of the YC2.3 calmodulin domain: D21A, D57A, D94A, D130A^81^.

### Microscopy and reporter expression analysis

Images to quantify *flp-5* and *flp-6* reporter expression in FLP and AFD respectively, were acquired in a Zeiss Axioplan2 fluorescence microscope, with a 40× (air, NA = 0.95) objective and constant illumination parameters. Data were analysed by (i) counting the number of neurons with detectable neuropeptide expression and dividing by the total number of neurons examined (fractions of AFD neurons expressing *flp-6* are reported in Fig. 4h and fraction of FLP neurons expressing *flp-5* are reported in Fig. 7g) and (ii) quantifying the signal among positive neurons using ImageJ as ‘*flp-6*/*flp-5* reporter intensity’ (Figs. 4g, 7f). This latter quantification was performed by defining regions of interest around identified cell bodies, and subtracting the background signal in the nearby worm body^54^.

### Statistical tests

D’Agostino & Pearson test (*p* < 0.01) was used to test normality of distributions. Comparisons giving significant effects (*p* < 0.05) with ANOVAs were followed by Bonferroni posthoc tests. Dunn’s test was used as non-parametric test whenever the normality assumption criterion was not fulfilled. All tests were two-tailed. In every figure, statistical significance is represented as ns, not significant, *p < 0.05 and **p < 0.01 for comparison with N2 15 fed or respective N2 controls and #*p* < 0.05 and ##*p* < 0.01 for indicated comparison. The χ2 and Fisher’s exact tests were performed for discrete event contingency comparisons and statistical significance is represented as ns not significant, +*p* < 0.05 and ++*p* < 0.01. *p* values are reported in Supplementary Data 7. Bimodal distribution was represented as violin plot and the rest of the data is represented as bar graph with individual datapoints overlaid.

### Transgene construction and transgenesis

DNA prepared with a GenElute HP Plasmid miniprep kit (Sigma) was microinjected in the gonad to generate transgenic lines according to a standard protocol^82^. We used a [*unc-122p*::*GFP*] (gift from Piali Sengupta; Addgene plasmid # 8937^83^) co-injection marker to identify transgenic animals. The concentration of co-marker and for expression plasmids in the injection mixes are indicated in the strain list.

### Promoter plasmids (multisitegateway slot 1)

Entry plasmids containing specific promoters were constructed by PCR from N2 genomic DNA with primers flanked with attB4 and attB1r recombination sites and cloned into pDONR-P4-P1R vector (Invitrogen) by BP recombination. Primer sequences were the following:

dg507 [slot1 Entry gcy-8p]

attB4gcy-8_F: ggggacaactttgtatagaaaagttgATAGCAAAGGGCGTCGATTATCT

attB1rgcy-8_R: ggggactgcttttttgtacaaacttgTTTGATGTGGAAAAGGTAGAATCGAA

dg867 [slot1 Entry egl-6p]

attB4egl-6_F: ggggacaactttgtatagaaaagttgATTTTCCAGAGAGAACAGAGTCC

attB1regl-6_R: ggggactgcttttttgtacaaacttgTTGCTGAAAAGCTGTCATTGTG

dg868 [slot1 Entry flp-5p]

attB4flp-5_F: ggggacaactttgtatagaaaagttgATCGAATTTGTCGCCGATCTGTTACA

attB1rflp-5_R: ggggactgcttttttgtacaaacttgTAGTTGCGAGGAATGACTGTTTCG

dg865 [slot1 Entry dmsr-1p]

attB4dmsr-1_F: ggggacaactttgtatagaaaagttgATCAGACGTCGTTGTGGAAGTAG

attB1rdmsr-1_R: ggggactgcttttttgtacaaacttgTTTTGTTTGCTGTTCCTCTGTTC

dg1015 [slot1 Entry lgc-39p]

attB4lgc-39_F: ggggacaactttgtatagaaaagttgATCGCTATCATCGTCTCCAAATCG

attB1rlgc-39_R: ggggactgcttttttgtacaaacttgTCGATGATTCACATCAGGGATGC

dg68 [slot1 Entry mec-3p] was created previously^84^.

### Coding sequence plasmids (multisitegateway slot 2)

Entry plasmids containing specific coding DNA sequences (cds) or genomic sequence (gs) were constructed by PCR from N2 cDNA or N2 genomic DNA with primers flanked with attB1 and attB2 recombination sites and cloned into pDONR_221 vector (Invitrogen) by BP recombination. Primer sequences were the following:

dg1014 [slot2 Entry flp-6cds]

attB1flp-6_F: ggggacaagtttgtacaaaaaagcaggctTAATGAACTCTCGTGGGTTGATTTTGA

attB2flp-6_R: ggggaccactttgtacaagaaagctgggtCTTATCGTCCGAATCTCATGTATGCT

dg872 [slot2 Entry egl-6gs]

attB1egl-6_F: ggggacaagtttgtacaaaaaagcaggctTAATGAATGACACACTGATCTGTACA

attB2egl-6_R: ggggaccactttgtacaagaaagctgggtCTTAAGACCCGACATATGAGCTTG

dg875 [slot2 Entry flp-5cds]

attB1flp-5_F: ggggacaagtttgtacaaaaaagcaggctTAATGAGCAGCCGAAGCACCAC

attB2flp-5_R: ggggaccactttgtacaagaaagctgggtCTTAGCCGAATCGGATGAATTTGGCT

dg871 [slot2 Entry dmsr-7cds]

attB1dmsr-7_F: ggggacaagtttgtacaaaaaagcaggctTAATGGAATGTCCGCACGATGC

attB2dmsr-7_R: ggggaccactttgtacaagaaagctgggtCTTAAAGTTGATGTTCTCTACTGCTG

dg869 [slot2 Entry dmsr-1Acds]

attB1dmsr-1a_F: ggggacaagtttgtacaaaaaagcaggctTAATGGAGTTTACCGAATGCAAAAC

attB2dmsr-1a_R: ggggaccactttgtacaagaaagctgggtCTCAAATGTTTTGAAAGTGTCCACG

dg870 [slot2 Entry dmsr-1Bcds]

attB1dmsr-1b_F: ggggacaagtttgtacaaaaaagcaggctTAATGGAGTTTACCGAATGCAAAAC

attB2dmsr-1b_R: ggggaccactttgtacaagaaagctgggtCTTATTTCCGTACTGTTTCTTCGTAC

dg88 [slot2 Entry TeTxcds] (aka pWD157) was a gift of Wayne Davis. The generation of dg651 [slot2 Entry egl-13NLS::wrmScarlet], of dg353 [slot2 Entry mNeongreen cds] and dg650 [slot2 Entry NLS_ceBFPcds] were described in^85,86^, and^54^ respectively.

### 3’ UTR and tagging plasmids (multi-site gateway slot3)

mg277 [SL2::mCherry] was created previously^84^. mg211 [EntrySlot3unc-54UTR] (aka pMH473) was a gift from Marc Hammarlund.

### Expression plasmids used for transgenesis

dg931 [gcy-8p::QF::unc-54UTR] was created through a LR recombination reaction between dg507, dg240, mg211, and pDEST-R4-P3.

dg899 [egl-6p::QF::unc-54UTR] was created through a LR recombination reaction between dg867, dg240, mg211, and pDEST-R4-P3.

dg243 [mec-3p::QF::unc-54UTR] was created through a LR recombination reaction between dg68, dg240, mg211, and pDEST-R4-P3.

dg898 [flp-5p::QF::unc-54UTR] was created through a LR recombination reaction between dg868, dg240, mg211, and pDEST-R4-P3.

dg900 [dmsr-1p::QF::unc-54UTR] was created through a LR recombination reaction between dg865, dg240, mg211, and pDEST-R4-P3.

dg1017 [lgc-39p::QF::unc-54UTR] was created through a LR recombination reaction between dg1015, dg240, mg211, and pDEST-R4-P3.

dg255 [QUASp::TeTxcds::SL2::mCherry] was created through a LR recombination reaction between dg229, dg88, mg277, and pDEST-R4-P3.

dg839 [QUASp::flp-6cds::SL2::mCherry] was created through a LR recombination reaction between dg229, dg827, mg277, and pDEST-R4-P3.

dg894 [QUASp::egl-6gs::SL2::mCherry] was created through a LR recombination reaction between dg229, dg872, mg277, and pDEST-R4-P3.

dg838 [QUASp::eat-4cds::SL2::mCherry] was created through a LR recombination reaction between dg229, dg713, mg277, and pDEST-R4-P3.

dg893 [QUASp::flp-5cds::SL2::mCherry] was created through a LR recombination reaction between dg229, dg875, mg277, and pDEST-R4-P3.

dg895 [QUASp::dmsr-7cds::SL2::mCherry] was created through a LR recombination reaction between dg229, dg871, mg277, and pDEST-R4-P3.

dg897 [QUASp::dmsr-1Acds::SL2::mCherry] was created through a LR recombination reaction between dg229, dg869, mg277, and pDEST-R4-P3.

dg896 [QUASp::dmsr-1Bcds::SL2::mCherry] was created through a LR recombination reaction between dg229, dg870, mg277, and pDEST-R4-P3.

dg373 [QUASp::mNeongreencds::unc-54UTR] was created through a LR recombination reaction between dg229, dg353, mg211, and pDEST-R4-P3.

dg996 [QUASp::wrmScarletcds::unc-54UTR] was created through a LR recombination reaction between dg229, dg353, mg211, and pDEST-R4-P3.

dg653 [mec-3p::NLS_CeBFPcds::unc-54UTR] was created through a LR recombination reaction between dg68, dg650, mg211, and pDEST-R4-P3.

dg1024 [QUASp::YC2.3(D21A, D57A, D94A, D130A)::unc-54UTR] was created by gene synthesis by introducing mutations in cameleon.

### *C. elegans* strains

A list of the strains used in the present study is provided in Supplementary Data 6^87–89^.

### Reporting summary

Further information on research design is available in the Nature Portfolio Reporting Summary linked to this article.

## Supplementary information

Supplementary Information

Description of Additional Supplementary Files

Supplementary Movie 1

Supplementary Movie 2

Supplementary Data 1

Supplementary Data 2

Supplementary Data 3

Supplementary Data 4

Supplementary Data 5

Supplementary Data 6

Supplementary Data 7

Reporting Summary

## Data Availability

The authors declare that the data supporting the findings of this study are available within the paper and its supplementary information files. Source data are provided with this paper.

## Source data

Source Data

## Code availability

Code for Monte-Carlo simulations is available in Supplementary Data 2.
