# Supplementary material for: Multisite regulation integrates multimodal context in sensory circuits to control persistent behavioral states in C. elegans
Source: Nat Commun. 2023 May 26;14:3052. doi: 10.1038/s41467-023-38685-1 (PMC10220067; doi:10.1038/s41467-023-38685-1)
Supplement: Supplementary file 1 — Supplementary Information [file 41467_2023_38685_MOESM1_ESM.pdf]

Multisite regulation integrates multimodal context in sensory circuits to control persistent behavioral states in *C. elegans*

SUPPLEMENTAL INFORMATION

Supplementary Figure 1

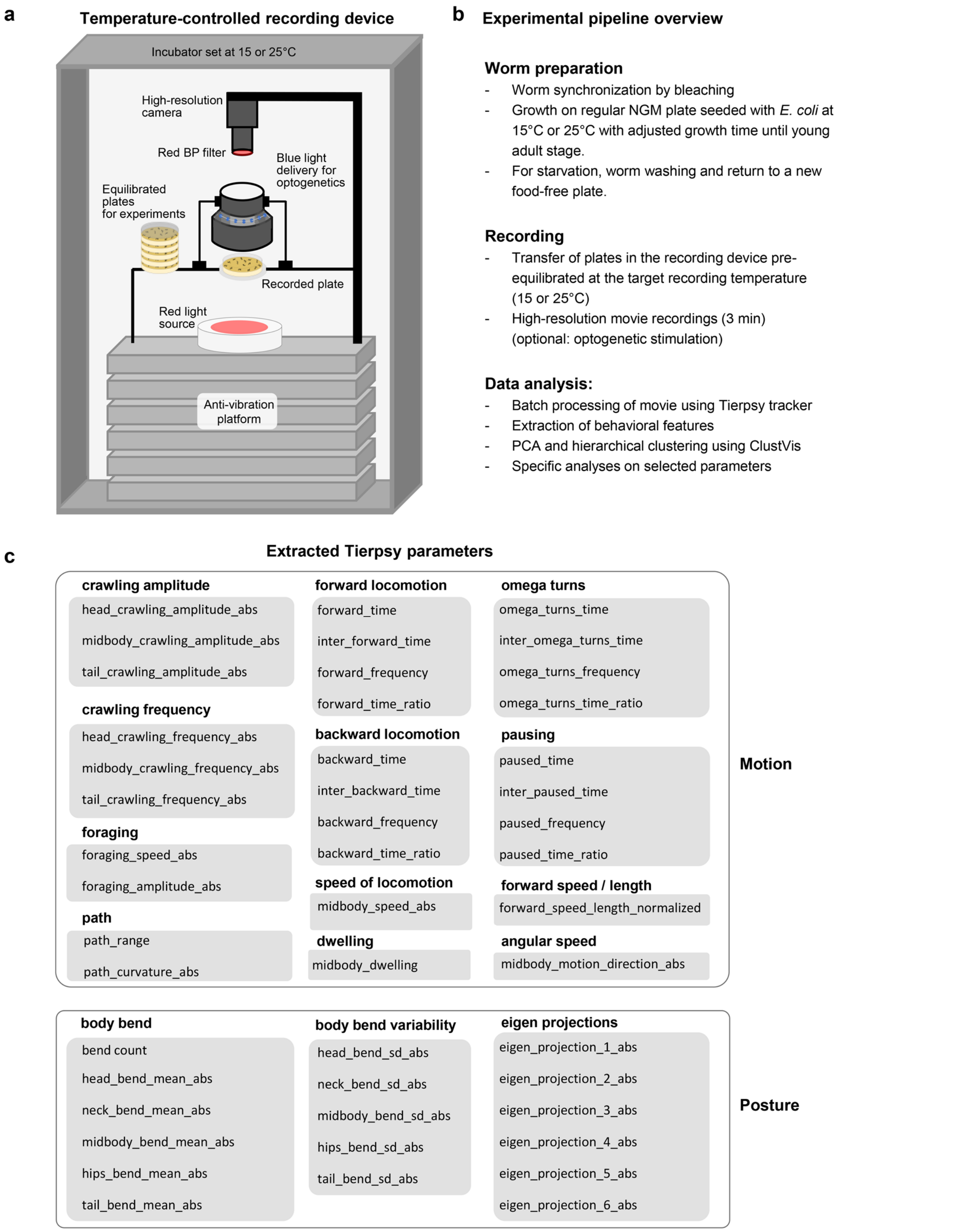

Supplementary Figure 1. Recording device, experimental pipeline overview and list of extracted behavioral parameters for motion and posture.

Schematic of the recording device used for behavioral recording in a tightly controlled thermal environment (**a**). The whole system is inside an incubator and isolated from the intrinsic vibration generated by the device via an anti-vibration platform (note that both the camera and the recorded plate are hence isolated). Overview of the experimental pipeline (**b**) and of the extracted Tierpsy tracker parameters (**c**).

Supplementary Figure 2

a

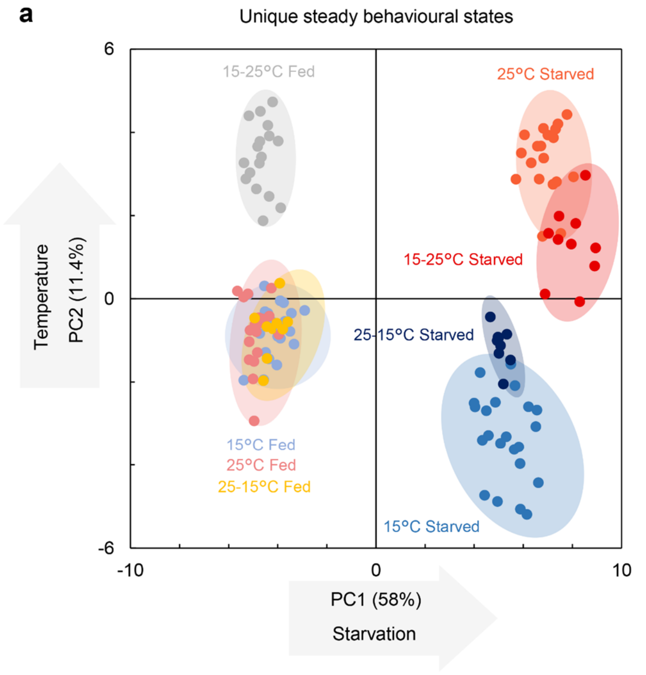

b

	15-25°C Fed	15°C Fed	25-15°C Fed	25°C Fed	25°C Starved	15-25°C Starved	15°C Starved	25-15°C Starved
15-25°C Fed	1.0E+00<br>1.0E+00	3.1E-01<br>7.2E-06	5.5E-01<br>2.3E-03	9.1E-01<br>1.9E-05	1.9E-05<br>1.0E+00	1.2E-03<br>4.6E-03	7.2E-06<br>7.2E-06	2.3E-03<br>2.3E-03
15°C Fed	3.1E-01<br>7.2E-06	1.0E+00<br>1.0E+00	1.0E+00<br>1.0E+00	1.7E-02<br>1.0E+00	2.8E-06<br>2.8E-06	5.0E-04<br>1.1E-03	8.1E-07<br>1.6E-06	1.1E-03<br>6.2E-01
25-15°C Fed	5.5E-01<br>2.3E-03	1.0E+00<br>1.0E+00	1.0E+00<br>1.0E+00	6.4E-02<br>1.0E+00	1.6E-03<br>1.6E-03	1.2E-02<br>2.4E-02	1.1E-03<br>1.6E-03	1.8E-02<br>8.2E-01
25°C Fed	9.1E-01<br>1.9E-05	1.7E-02<br>1.0E+00	6.4E-02<br>1.0E+00	1.0E+00<br>1.0E+00	8.2E-06<br>8.2E-06	8.1E-04<br>3.5E-03	2.8E-06<br>1.5E-05	1.6E-03<br>8.4E-01
25°C Starved	1.9E-05<br>1.0E+00	2.8E-06<br>2.8E-06	1.6E-03<br>1.6E-03	8.2E-06<br>8.2E-06	1.0E+00<br>1.0E+00	5.7E-02<br>1.1E-02	6.9E-05<br>2.8E-06	1.6E-03<br>1.6E-03
15-25°C Starved	1.2E-03<br>4.6E-03	5.0E-04<br>1.1E-03	1.2E-02<br>2.4E-02	8.1E-04<br>3.5E-03	5.7E-02<br>1.1E-02	1.0E+00<br>1.0E+00	5.0E-04<br>5.0E-04	1.2E-02<br>1.2E-02
15°C Starved	7.2E-06<br>7.2E-06	8.1E-07<br>1.6E-06	1.1E-03<br>1.6E-03	2.8E-06<br>1.5E-05	6.9E-05<br>2.8E-06	5.0E-04<br>5.0E-04	1.0E+00<br>1.0E+00	1.0E+00<br>1.6E-03
25-15°C Starved	2.3E-03<br>2.3E-03	1.1E-03<br>6.2E-01	1.8E-02<br>8.2E-01	1.6E-03<br>8.4E-01	1.6E-03<br>1.6E-03	1.2E-02<br>1.2E-02	1.0E+00<br>1.6E-03	1.0E+00<br>1.0E+00

Supplementary Figure 2. Distinction between behavioral states 6 hours after food and temperature-dependent behavioral transitions

Summary of the main steady behavioral states adopted by animals in various thermal and feeding contexts after 6 h of the indicated thermal and/or feeding shifts. Overlaid dataset from Figure 1C, 1F, 1I and 1L, showing all replicates for each condition (individual dots) and corresponding 95% CI (colored ellipses) (**a**). Each data point represents the average value for 3-min recordings on at least 40 worms. Two-sided Dwass-Steel-Critchlow-Fligner pairwise comparisons table for the eight conditions on PC1 and PC2 (**b**). *p*-values for PC1 (top value in each cell) and PC2 (bottom value). Conditions presenting only non-significant *p*-values (>.05) with each other were clustered together and highlighted with a grey shade. Source data are provided as a Source Data file.

Supplementary Figure 3

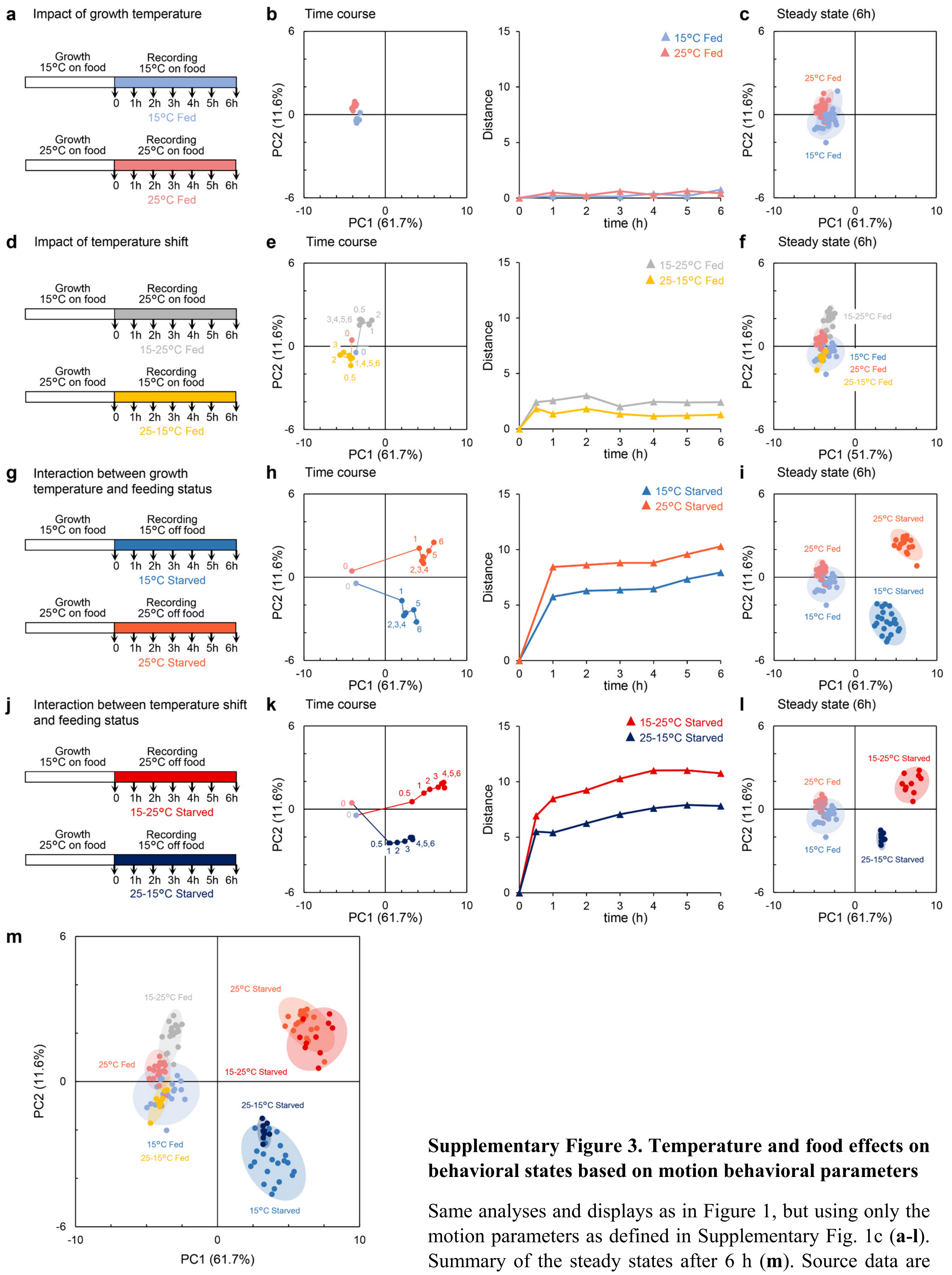

Supplementary Figure 3. Temperature and food effects on behavioral states based on motion behavioral parameters

Same analyses and displays as in Figure 1, but using only the motion parameters as defined in Supplementary Fig. 1c (**a-l**). Summary of the steady states after 6 h (**m**). Source data are provided as a Source Data file.

Supplementary Figure 4

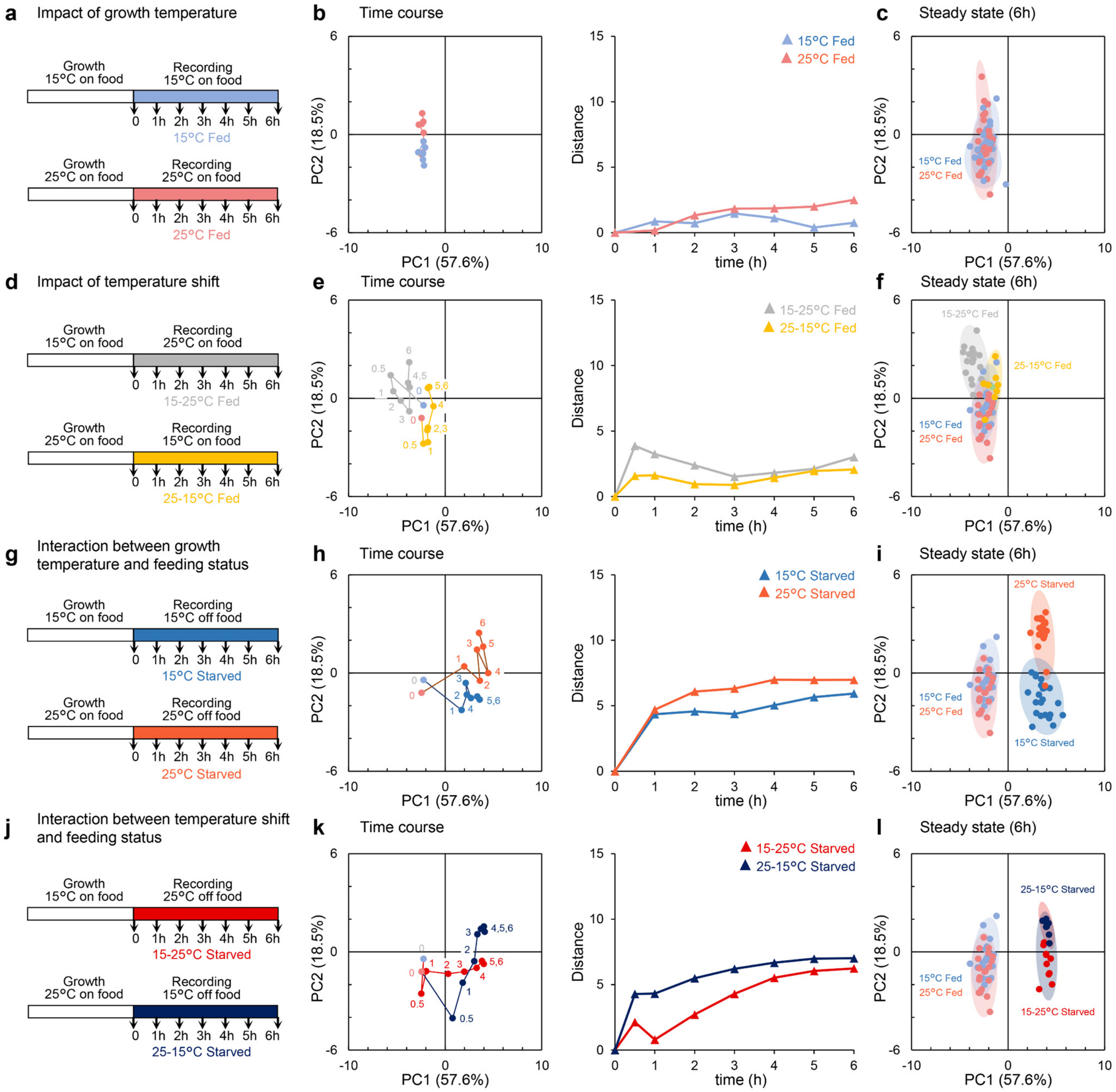

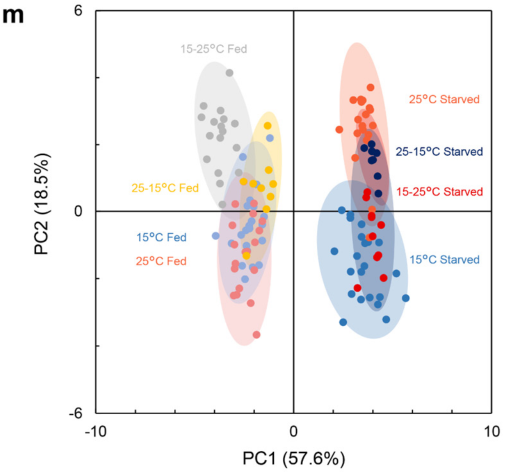

Supplementary Figure 4. Temperature and food effects on behavioral states based on postural behavioral parameters

Same analyses and displays as in Figure 1, but using only the postural parameters as defined in Supplementary Fig. 1c (**a-l**). Summary of the steady states after 6 h (**m**). Source data are provided as a Source Data file.

Supplementary Figure 5

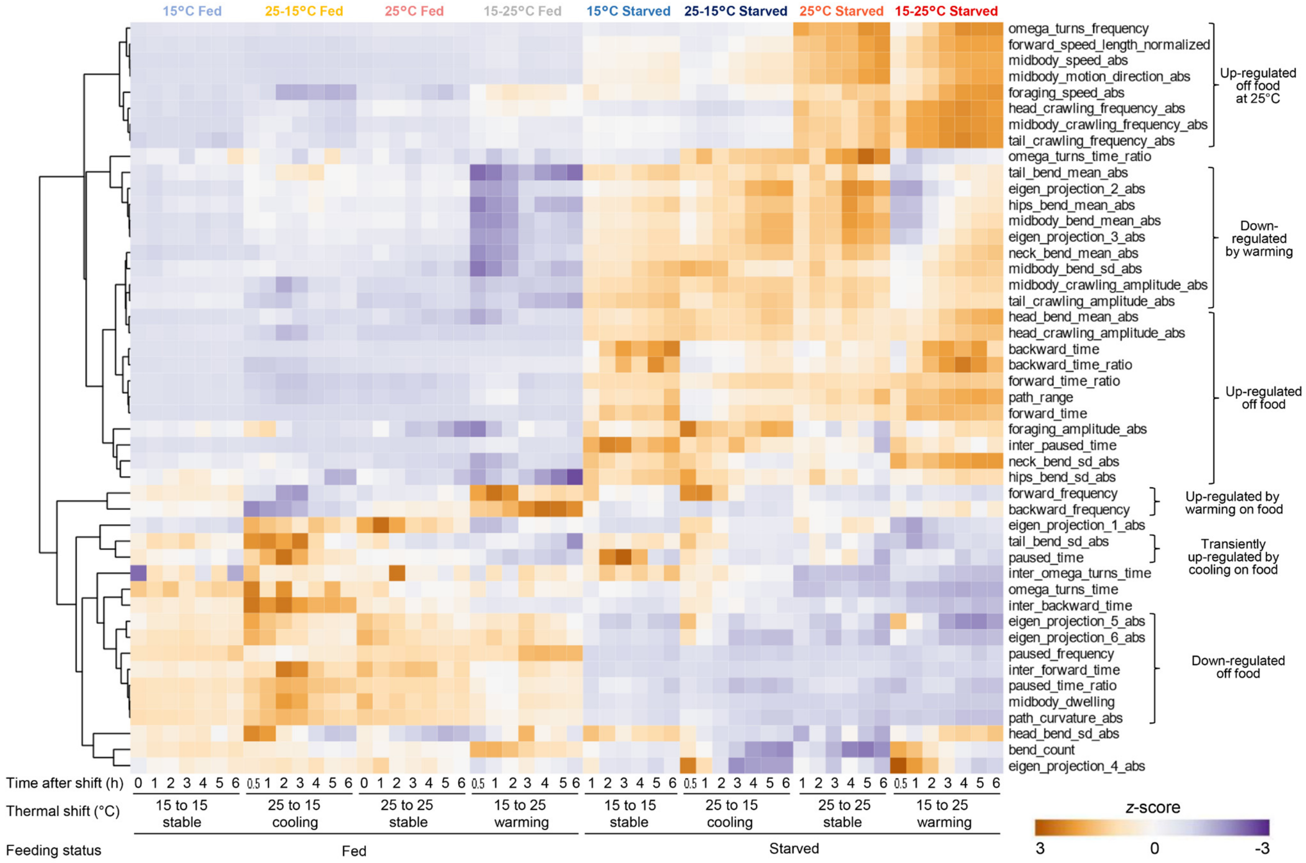

Supplementary Figure 5. Behavioral codes during food and temperature-dependent behavioral transitions

Heat-map of behavioral parameters of *z*-scores across the indicated conditions and hierarchical clustering based on Euclidian distance (tree on the left). Clusters of parameters affected by starvation, growth temperature and/or recent temperature shift are annotated (brackets on the right). A time series over 6 h is presented for each condition based on the same data set as the one use for PCA analyses reported in Fig. 1. Each data point represents the average value for 3-min recordings on at least three independent worm populations (≥40 worms each). Source data are provided as a Source Data file.

Supplementary Figure 6

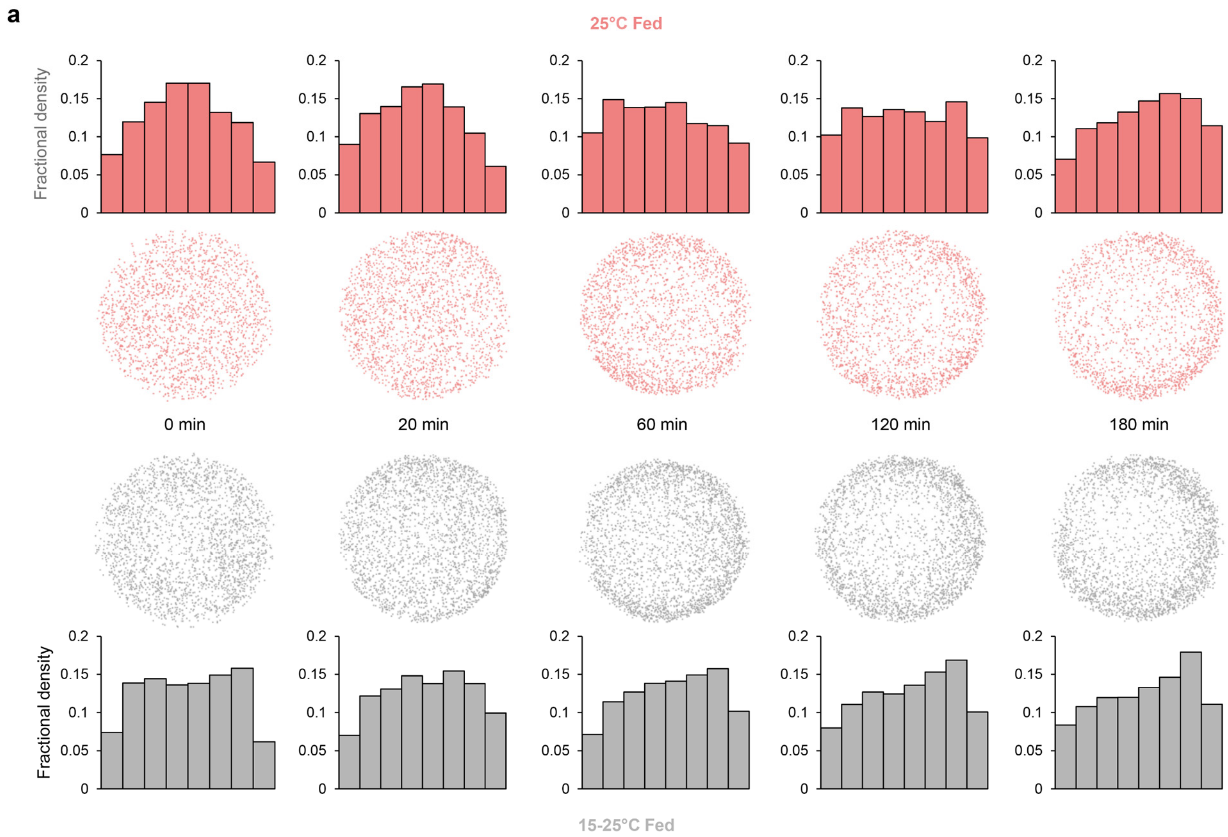

Supplementary Figure 6. Distribution of worms during thermotaxis assay

Histograms of on-food thermotaxis assays in wild type animals revealing a faster thermotactic movement toward recent growth temperature in scanning animals (6h after warming) as compared to dwelling animals held at 25°C as shown in Fig. 2d-f. n =10 assays each with ≥50 worms. Source data are provided as a Source Data file.

Supplementary Figure 7

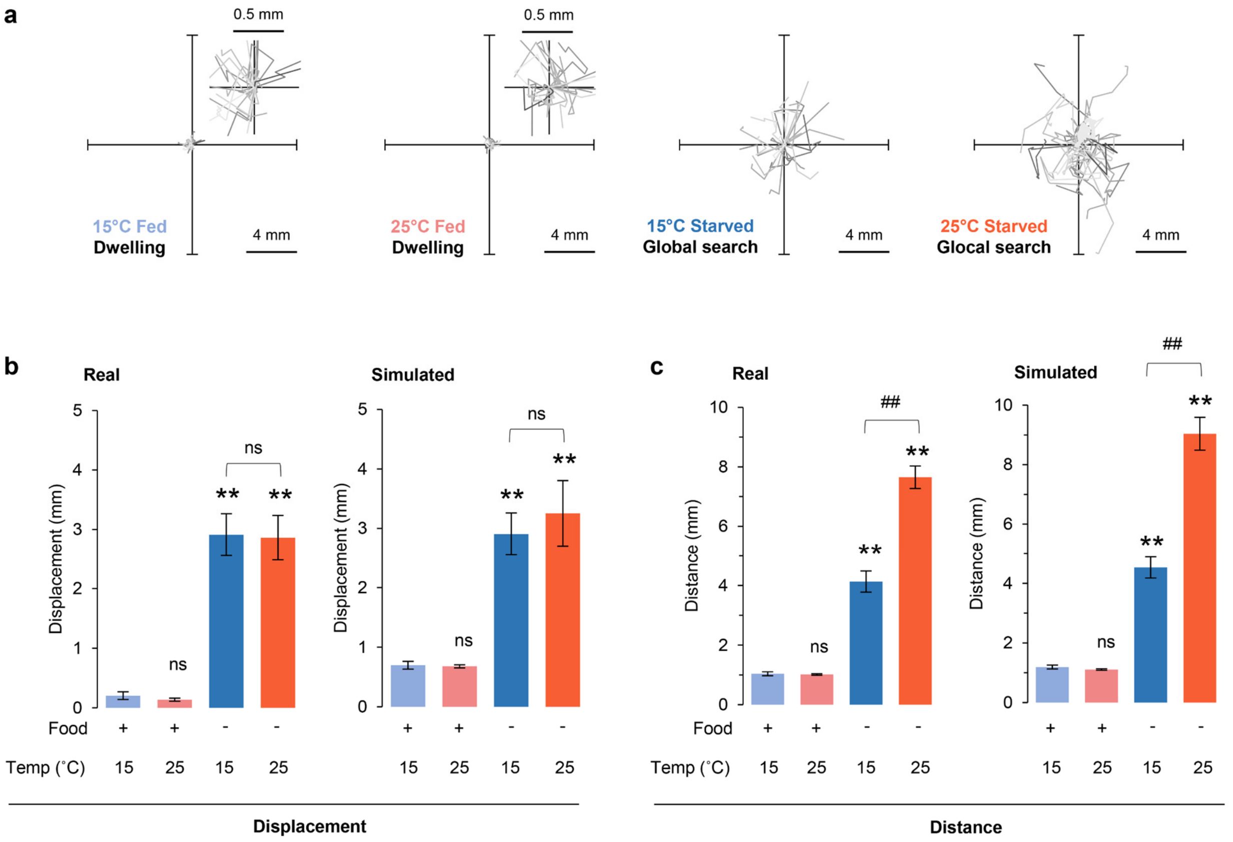

Supplementary Figure 7. Dispersal trajectory simulations comparing dwelling, global and glocal search states

Results of Monte-Carlo simulations considering the average frequency of turns and speed measured in worm populations in isothermal environments. Fifteen 1-min trajectories for each condition (**a**). For the sake of better visibility, dwelling trajectories are magnified (insets). Average (± s.e.m,) for animal displacement (**b**, corresponding to how far animals moved from their starting point) and covered distance (**c**, corresponding to the path length of each track). Both simulated and real data are presented side-by-side. Real data are the same as in Fig. 2b and c. **, p<.01 versus 15°C Fed condition, ##, p<.01 versus the indicated control by Bonferroni posthoc tests. ns, not significant. For simulations: data as mean ± s.e.m. of n= 200 independent simulated worm trajectories per condition. Source data are provided as a Source Data file.

Supplementary Figure 8

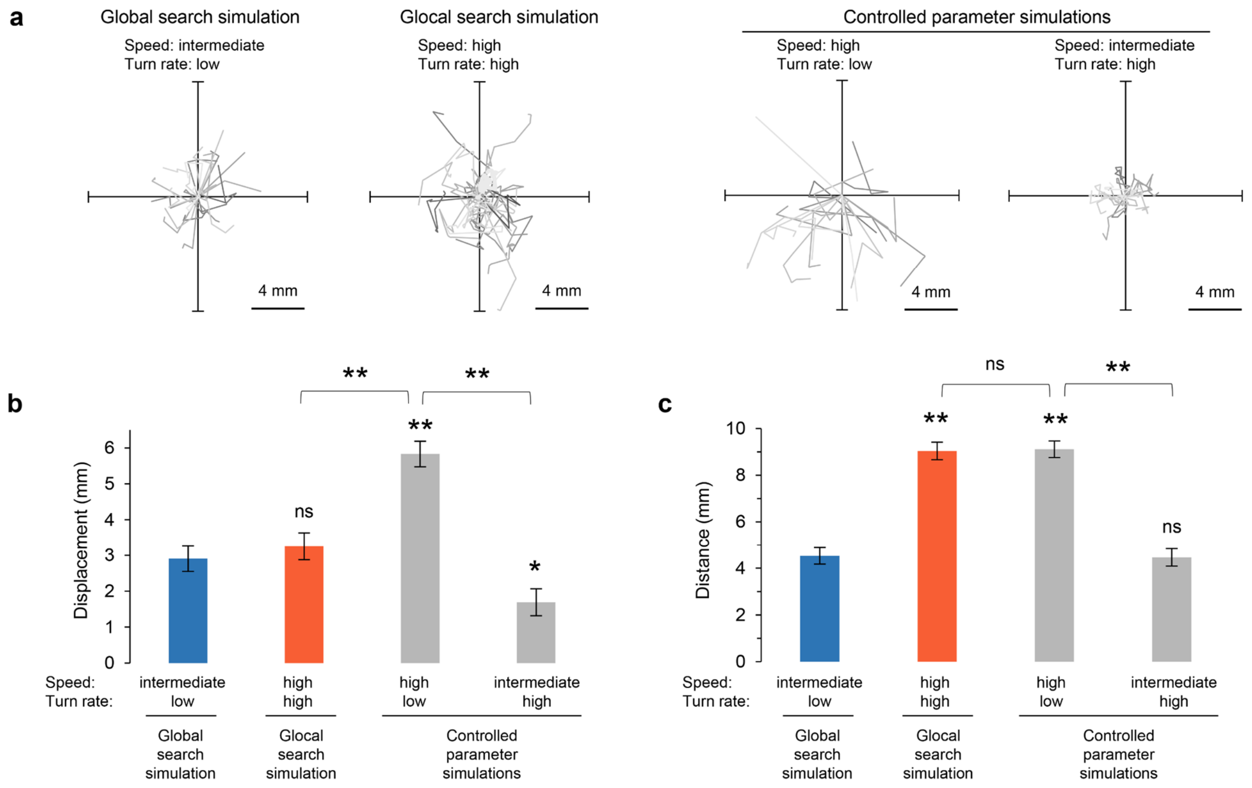

Supplementary Figure 8. Dissection of global search and glocal search dispersal trajectories with controlled parameter simulations

Results of Monte-Carlo simulations considering the average frequency of turns and speed measured in worm populations in isothermal environments. Fifteen 1-min trajectories for each condition (**a**). Average (± s.e.m) for animal displacement (**b**, corresponding to how far animals moved from their starting point) and covered distance (**c**, corresponding to the path length of each track). Data for global search and glocal search (same as in Supplementary Fig. 7) are presented together with controlled parameter simulation, in which only one parameter was changed at a time (either only turning rate or only speed). Data as mean ± s.e.m. of n= 200 independent simulated worm trajectories per condition (**b**, **c**). Source data are provided as a Source Data file.

Supplementary Figure 9

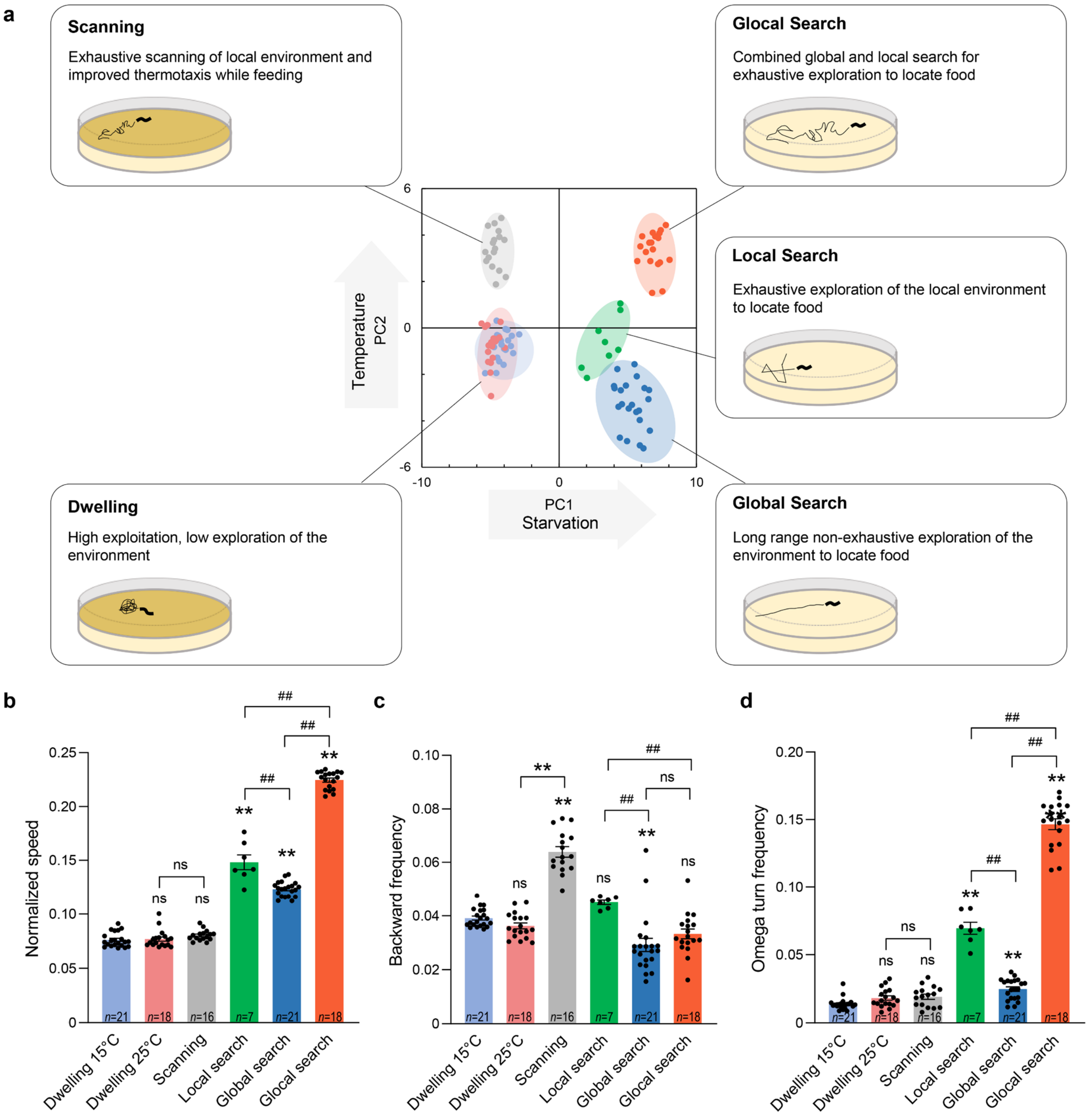

Supplementary Figure 9. Global or glocal search states are distinct from the local search state observed 5 min after food deprivation. PCA analysis presented like in Fig. 1, showing that the Local search state after 5 min of food deprivation locates to a distinct location in the PCA space as compared to Dwelling, Scanning, Global search or Glocal search states (**a**). Specific behavioral states are associated with specific values for speed (**b**), backward frequency (**c**) and omega turn frequency (**d**). Data as mean ± s.e.m.; indicated *n* correspond to independent assays, each scoring ≥30 worms (**b**, **c**, **d**). **, p<.01 versus N2 with the same treatment by Bonferroni post-hoc tests. ns, not significant. Source data are provided as a Source Data file.

Supplementary Figure 10

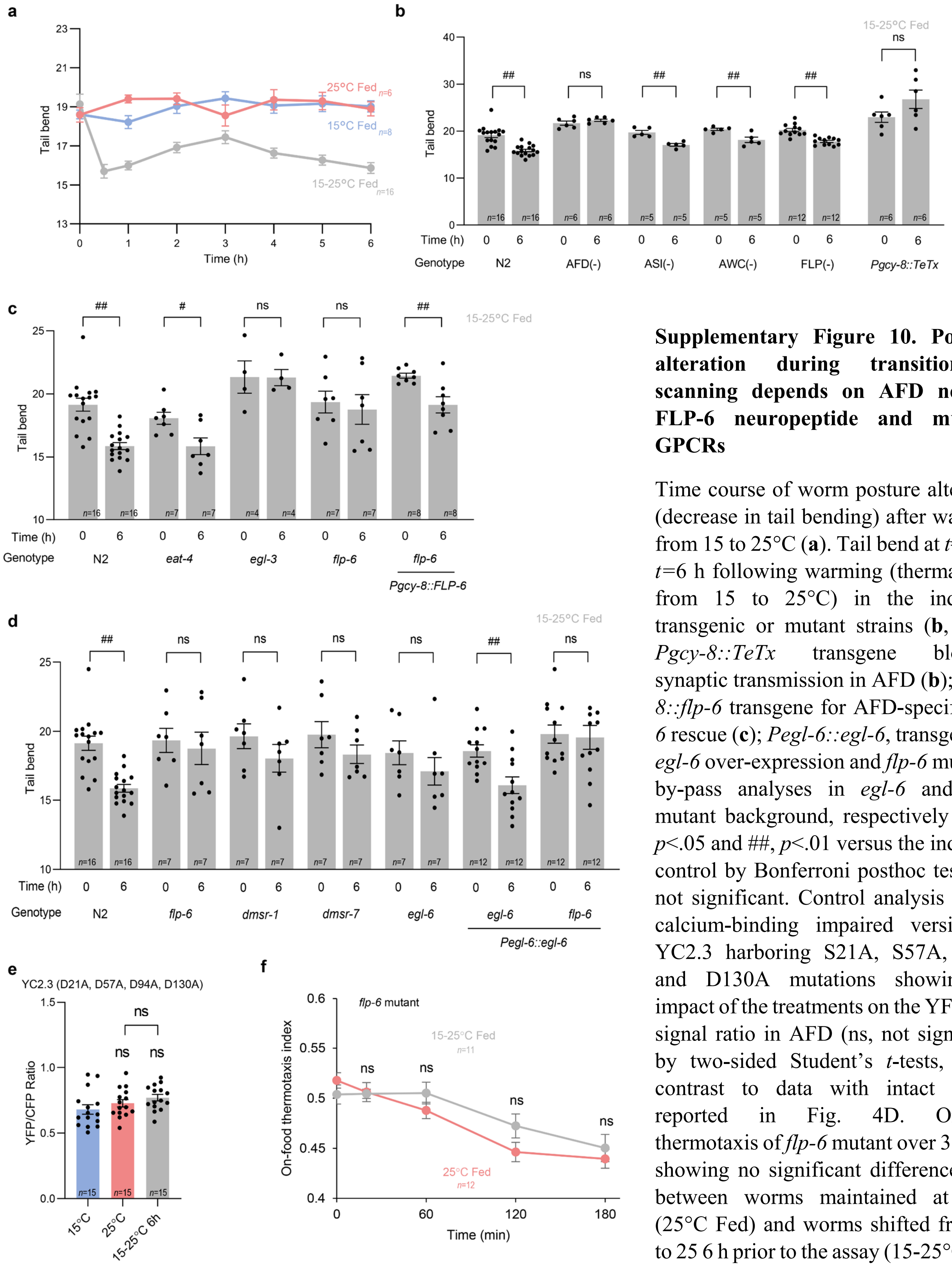

Supplementary Figure 10. Postural alteration during transition to scanning depends on AFD neuron, FLP-6 neuropeptide and multiple GPCRs

Time course of worm posture alteration (decrease in tail bending) after warming from 15 to 25°C (**a**). Tail bend at t=0 and t=6 h following warming (thermal shift from 15 to 25°C) in the indicated transgenic or mutant strains (**b**, **c**, **d**). *Pgcy-8::TeTx* transgene blocking synaptic transmission in AFD (**b**); *Pgcy-8::flp-6* transgene for AFD-specific *flp-6* rescue (**c**); *Pegl-6::egl-6*, transgene for *egl-6* over-expression and *flp-6* mutation by-pass analyses in *egl-6* and *flp-6* mutant background, respectively (**d**). # $p<.05$ and ##, $p<.01$ versus the indicated control by Bonferroni posthoc tests. ns, not significant. Control analysis with a calcium-binding impaired version of YC2.3 harboring S21A, S57A, D94S and D130A mutations showing no impact of the treatments on the YFP/CFP signal ratio in AFD (ns, not significant by two-sided Student's t-tests, **e**), in contrast to data with intact YC2.3 reported in Fig. 4D. On-food thermotaxis of *flp-6* mutant over 3 hours, showing no significant differences (ns) between worms maintained at 25°C (25°C Fed) and worms shifted from 15 to 25 6 h prior to the assay (15-25°C Fed) (**f**). Data as mean ± s.e.m.; indicated n correspond to independent assays, each scoring ≥30 worms (**a**, **b**, **c**, **d**, **f**) or independent neurons (**e**). Source data are provided as a Source Data file.

Supplementary Figure 11

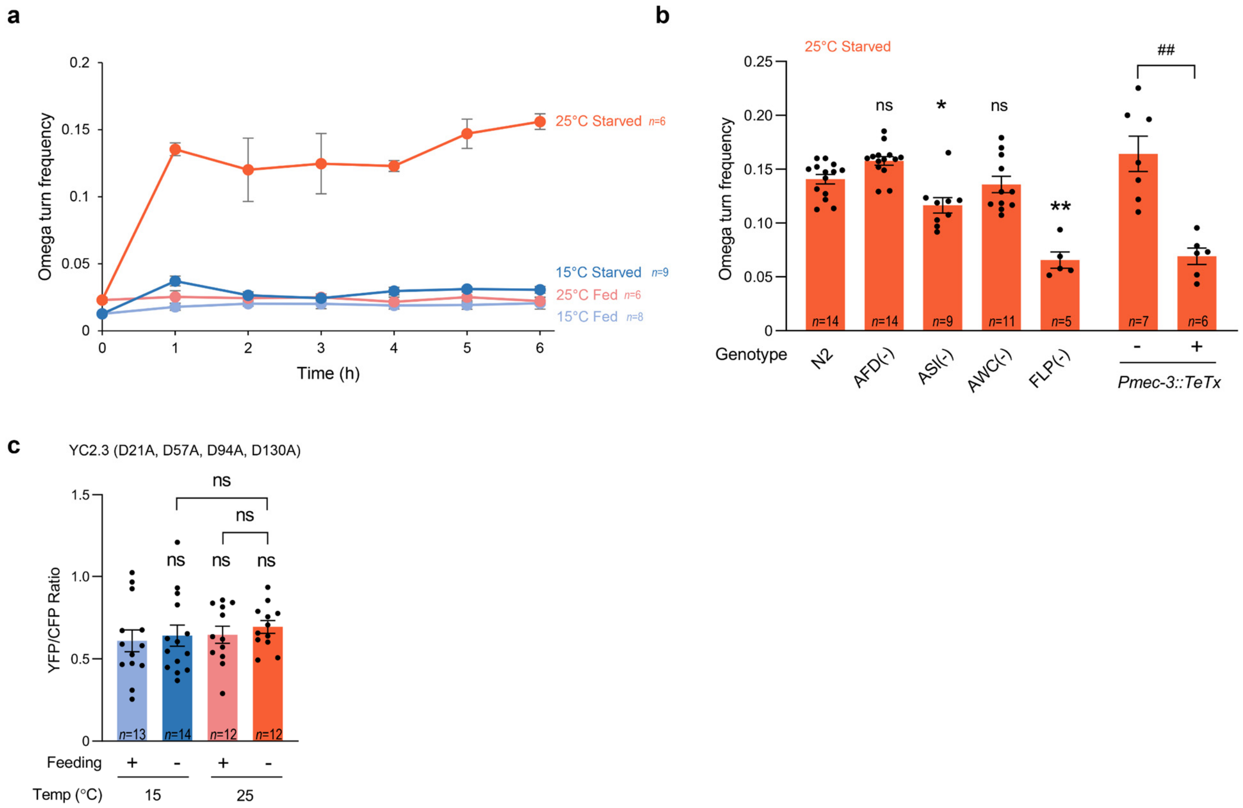

Supplementary Figure 11. FLP thermosensory neurons are essential for omega turn increase during glocal search

Time course of omega turn frequency increase after starvation at 25°C (25°C Starved) as compared to controls (15°C Fed, 25°C Fed, 15°C Starved) (**a**). Omega turn frequency measured after 6 h of starvation at 25°C in wild type (N2), in transgenic lines with genetic ablation of the indicated neurons, or in animals carrying a *Pmec-3::TeTx* transgene blocking neurotransmission in FLP (**b**). Like for speed elevation (Fig. 5), FLP plays a major role in the up-regulation of omega turns. Control analysis with a calcium-binding impaired version of YC2.3 harboring S21A, S57A, D94S and D130A mutations showing no impact of the treatments on the YFP/CFP signal ratio in FLP (ns, not significant by two-sided Student's *t*-tests, **c**), in contrast to data with intact YC2.3 reported in Fig. 5D. Data as mean ± s.e.m.; indicated *n* correspond to independent assays, each scoring ≥30 worms (**a**, **b**), or independent neurons (**c**). Source data are provided as a Source Data file.

Supplementary Figure 12

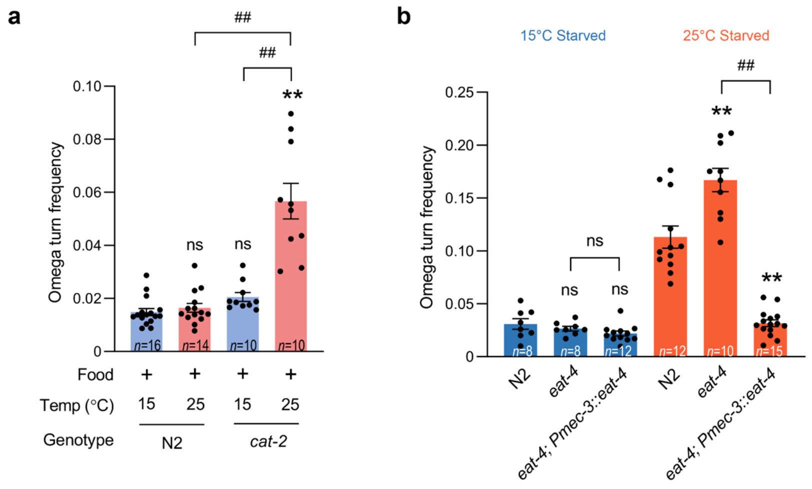

Supplementary Figure 12. Dopamine and glutamate signaling controls state-dependent omega turns

Impact of a *cat*-2 mutation blocking dopamine biosynthesis on the omega turn frequency in fed animals at 15 or 25°C (**a**). Impact of *eat-4* mutation affecting glutamatergic signaling on the omega turn frequency in starved animals at 15 or 25°C (**b**). Data as mean ± s.e.m.; indicated *n* correspond to independent assays, each scoring ≥30 worms (**a**, **b**). **, *p*<.01 versus N2; ##, *p*<.01 versus the indicated condition by Bonferroni posthoc tests. ns, not significant. Source data are provided as a Source Data file.

Supplementary Figure 13

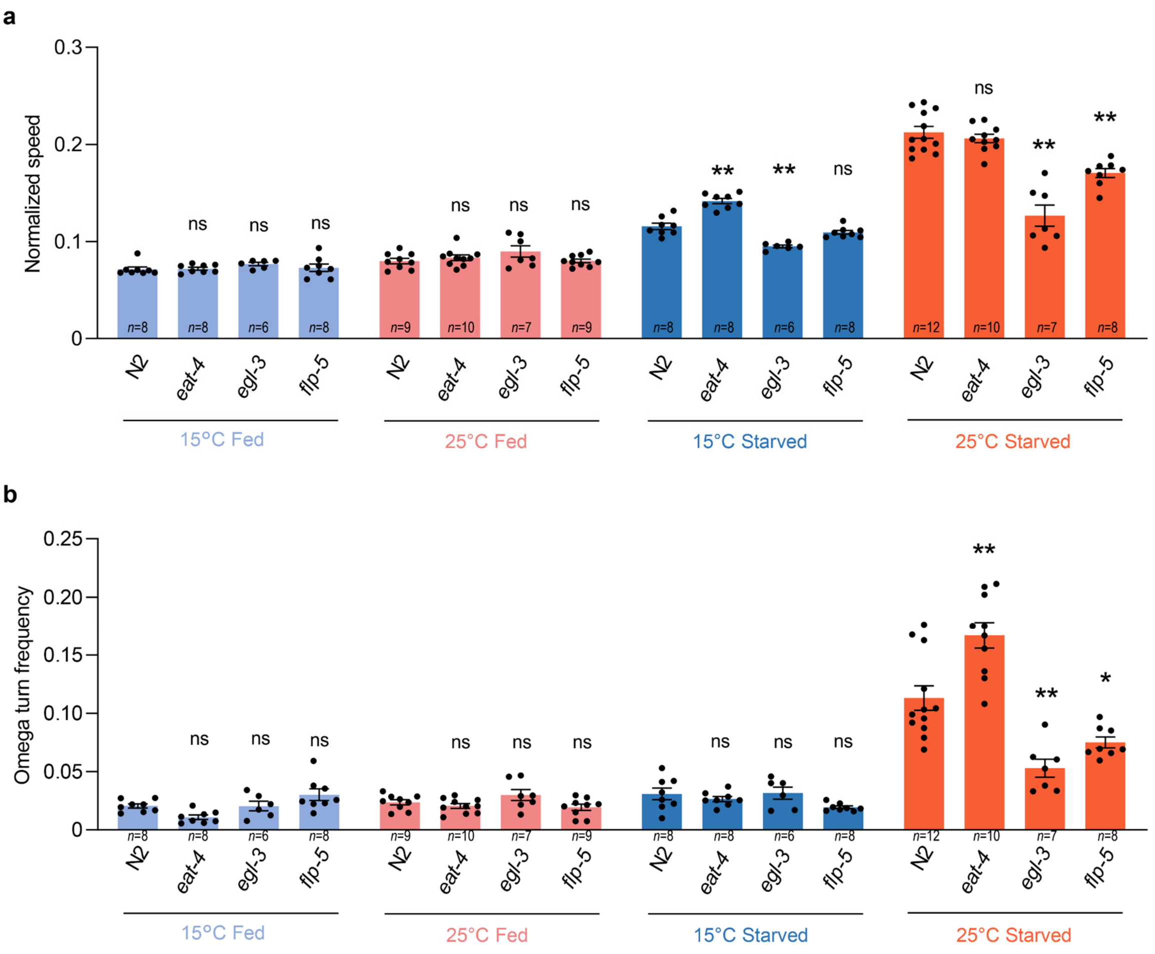

Supplementary Figure 13. Impact of *eat-4*, *egl-3* and *flp-5* mutations on speed and omega turn across feeding and temperature conditions. Control data for the speed analysis presented in Fig. 6e and 7a (**a**) and the omega turn analyses presented in Supplementary Figures 10b and 11a (**b**), and showing all four conditions (15°C Fed, 25°C Fed, 15°C Starved and 25°C Starved). Data as mean ± s.e.m.; indicated *n* correspond to independent assays, each scoring ≥30 worms (**a**, **b**). **, *p*<.01 versus N2 with the same treatment by Bonferroni post-hoc tests. ns, not significant. Source data are provided as a Source Data file.

Supplementary Figure 14

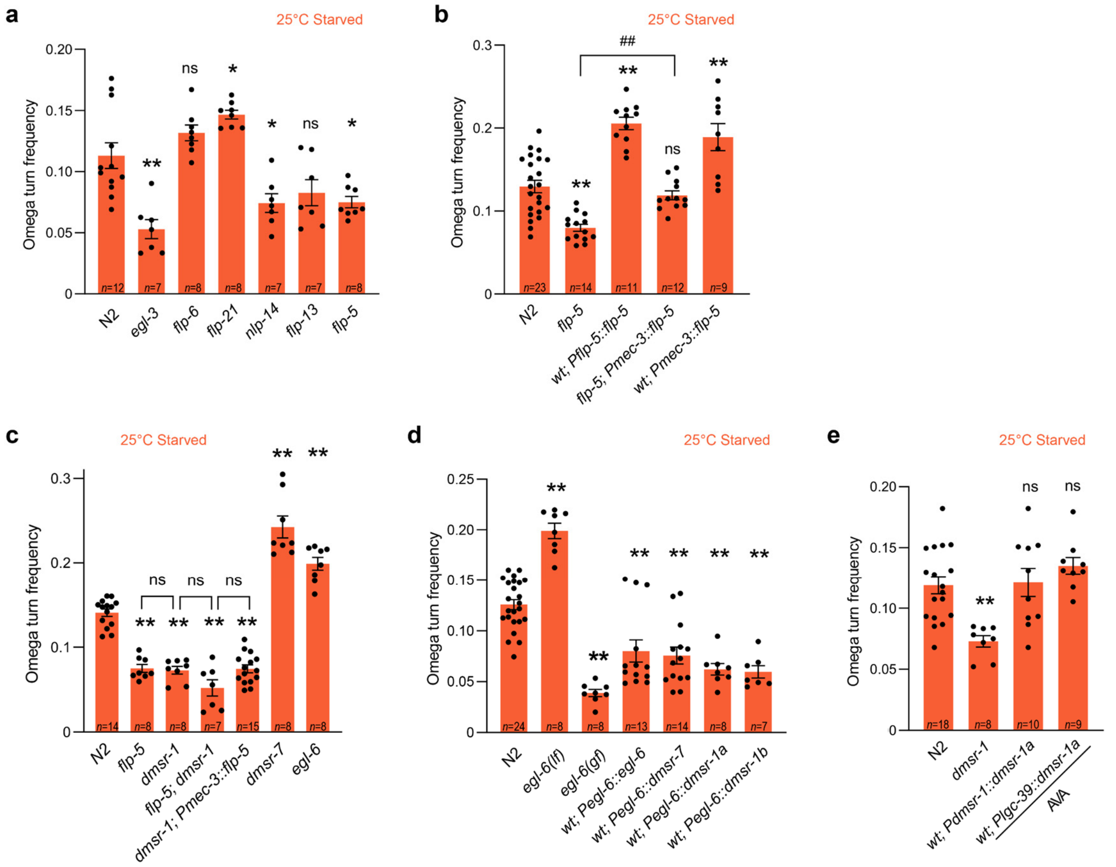

Supplementary Figure 14. Omega turn up-regulation during glocal search involves FLP-5/DMSR-1 signaling from FLP

Genetic dissection of the molecular signaling controlling omega turn frequency increase during glocal search. Data as mean ± s.e.m.; indicated *n* correspond to independent assays, each scoring ≥30 worms. Impact of neuropeptide-affecting mutations on the omega turn frequency of starved animals held at 25°C (**a**). Impact of *flp-5* mutation, over-expression with a *Pflp-5::flp-5* transgene, and rescue/over-expression with *Pmec-3::flp-5* transgene expressed in FLP (**b**). Impact of mutations affecting FLP-5 and its receptors (**c**). Impact of gain-of-function (gf) and loss-of-function (lf) mutations in *egl-6*, as well as FLP-5 receptor over-expression in *egl-6*-expressing cells, revealing that EGL-6, DMSR-1a, DMSR-1b and DMSR-7 have a similar inhibitory effect on omega turn frequency in starved animals at 25°C (**d**). No effect of DMSR-1 overexpression in *dmsr-1*-expressing cells or AVA-specific overexpression, respectively, on omega turn frequency in starved animals at 25°C (**e**). **, $p<.01$ versus N2; ##, $p<.01$ versus the indicated condition by Bonferroni posthoc tests. ns, not significant. Source data are provided as a Source Data file.

Supplementary Figure 15

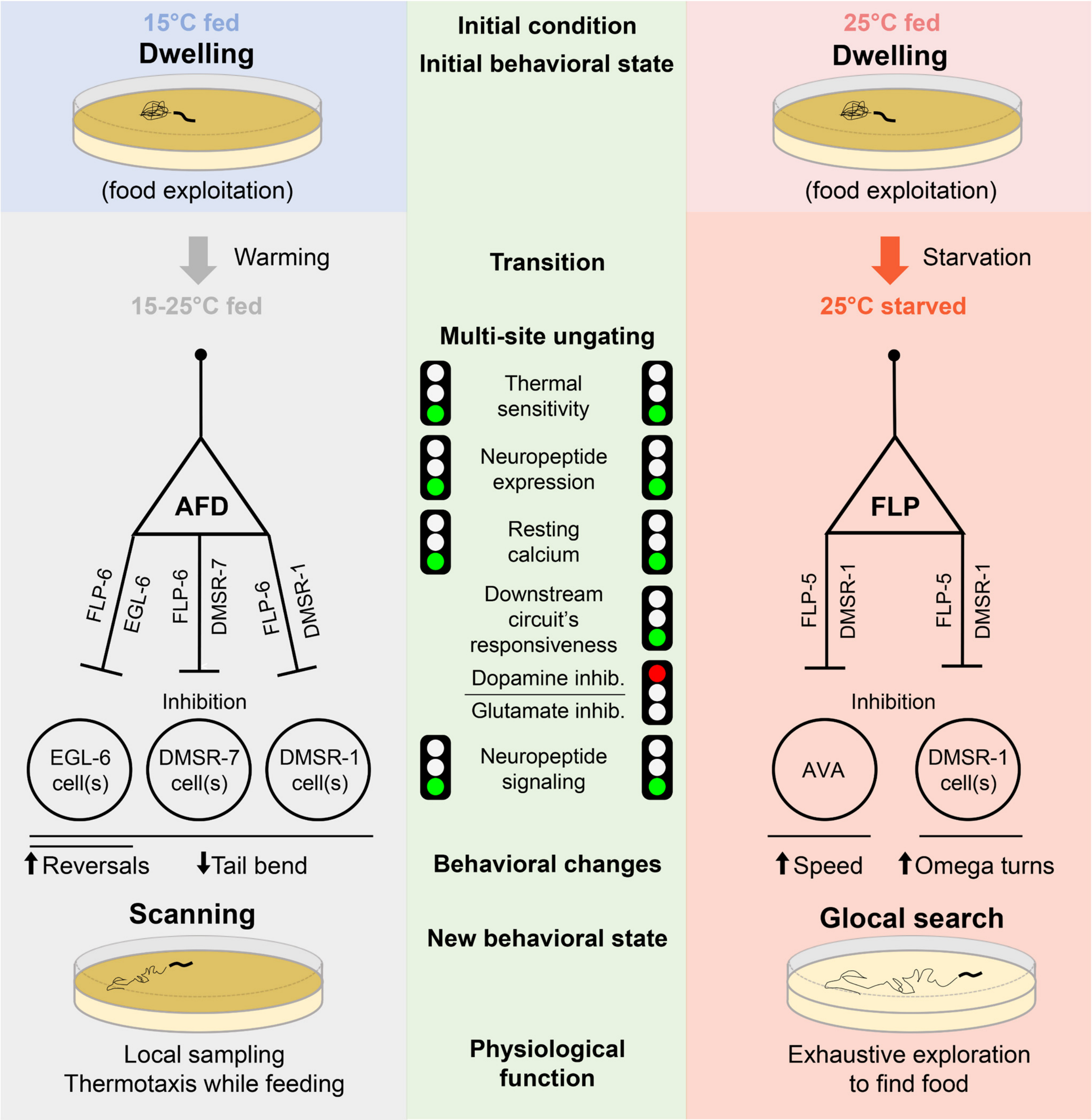

Supplementary Figure 15. Models of the multiple regulatory processes controlling behavioral state transitions from dwelling to scanning and glocal search, respectively.
