# Supplementary material for: Multisite regulation integrates multimodal context in sensory circuits to control persistent behavioral states in C. elegans
Source: Nat Commun. 2023 May 26;14:3052. doi: 10.1038/s41467-023-38685-1 (PMC10220067; doi:10.1038/s41467-023-38685-1)
Supplement: Supplementary file 2 — Description of Additional Supplementary Files [file 41467_2023_38685_MOESM2_ESM.pdf]

Description of Additional Supplementary Files

File Name: Supplementary Movie 1
Description: Representative movie of animals in dwelling versus scanning behavioral states

File Name: Supplementary Movie 2
Description: Representative movie of animals in global versus glocal search behavioral states

File Name: Supplementary Data 1
Description: Tracking parameter description

File Name: Supplementary Data 2
Description: Monte-Carlo simulation source code

File Name: Supplementary Data 3
Description: Tracking raw data on 47 parameters for all conditions

File Name: Supplementary Data 4
Description: PCA loadings

File Name: Supplementary Data 5
Description: PCA scores

File Name: Supplementary Data 6
Description: List of *C. elegans* strains

File Name: Supplementary Data 7
Description: *p* values for statistical tests reported in the Figures.
