# Supplementary material for: Multisite regulation integrates multimodal context in sensory circuits to control persistent behavioral states in C. elegans
Source: Nat Commun. 2023 May 26;14:3052. doi: 10.1038/s41467-023-38685-1 (PMC10220067; doi:10.1038/s41467-023-38685-1)
Supplement: Supplementary file 10 — Supplementary Data 6 [file 41467_2023_38685_MOESM10_ESM.docx]

**Strain list**

| **Strain Name** | **Genotype** | **Comments** |
| --- | --- | --- |
| N2 | Wild type | Wild type (WT) |
| GN112 | *pgIs2 [gcy-8p::TU#813 + gcy-8p::TU#814 + unc-122p::GFP + gcy-8p::mCherry + gcy-8p::GFP + ttx-3p::GFP]* | AFD ablation (gift from Miriam B. Goodman) ^87^. Fig. 4B, 5B, Supplementary Fig. 10B, 11B |
| PY7505 | *gpa-4p::TU318; gcy-27p::TU814; gcy-27p::GFP coelp::dsRED* | ASI ablation (Gift from Piali Sengupta) ^88^.  Fig. 4B, 5B, Supplementary Fig. 10B, 11B |
| PY7502 | *ceh-36delp::TU#813; ceh-36delp::TU#814; srtx-1p::gfp; Coelomycte::dsRED* | AWC ablation (Gift from Piali Sengupta) ^88^.  Fig. 4B, 5B, Supplementary Fig. 10B, 11B |
| DAG340 | *domEx303[mec‐3p::CZcasp3, flp‐4p::QF, QUAS::casp3NZ::SL2mCherry, unc‐122p::RFP]* | FLP ablation ^84^.  Fig. 4B, 5B, Supplementary Fig. 10B, 11B |
| DAG1642-1644 | *domEx1642-1644 [gcy-8p::QF 20 ng/ul, QUASp::TeTxcds::SL2mcherry 40 ng/ul, unc122p::GFP 20 ng/ul]* | Tetnus toxin expression with *gcy-8* promoter driving TeTx coding sequence (CDS).  Fig. 4B, Supplementary Fig. 10B |
| DAG1513 | *domEx1513[ttx-1prom::QF, QUAS::YC2.3]* | Expression of YC2.3 cameleon in AFD ^54^.  Fig. 4C, D, E |
| DAG1682 | *oyIs17(pgcy-8:GFP); domEx1682 [flp-6p::QF 10 ng/ul, QUASp::wrmScarlet 10 ng/ul]* | *flp-6* transcriptional reporter.  Fig. 4G, H |
| MT6308 | *eat-4(ky5) III* | Obtained from CGC.  Fig. 4F, 6E, Supplementary Fig. 10C, 12B, 13A, 13B |
| VC461 | *egl-3(gk238) V* | Obtained from CGC.  Fig. 4F, 7A, Supplementary Fig. 10C, 14A, 13A, 13B |
| VC2324 | *flp-6(ok3056) V* | Obtained from CGC.  Fig. 4, 7A, Supplementary Fig. 10C, 10D, 14A |
| DAG1648-1649 | *flp-6(ok3056) V; domEx1648-1649 [gcy-8p::QF 20 ng/ul, QUASp::flp-6cds::SL2mcherry 20 ng/ul, unc122p::GFP 20 ng/ul]* | *flp-6* rescue with *gcy-8* promoter driving *flp-6* coding sequence (cds).  Fig. 4F, Supplementary Fig. 10C |
| NQ915 | *dmsr-1(qn45) V* | Obtained from CGC.  Fig. 4I, 7H, 7L Supplementary Fig. 10D, 14C, 14E |
| DAG37 | *dmsr-7(syb2359)* | 1202bp deletion mutation made by genome editing (SunyBiotech, China).  Fig. 4I, 7H, Supplementary Fig. 10D, 14C |
| MT14666 | *egl-6(n4537) X* | Obtained from CGC.  Fig. 4I, 7H, 7K, Supplementary Fig. 10D, 14C, 14D |
| DAG1712-1713 | *egl-6(n4537) X; domEx1712-1713 [egl-6p::QF 30 ng/ul, QUASp::egl-6gs::SL2mcherry 30 ng/ul, unc122p::GFP 20 ng/ul]* | *egl-6* rescue with *egl-6* promoter driving *egl-6* genomic sequence (gs).  Fig. 4I, Supplementary Fig. 10D |
| DAG1716-1717 | *flp-6(ok3056) V; domEx1716-1717 [egl-6p::QF 30 ng/ul, QUASp::egl-6gs::SL2mcherry 30 ng/ul, unc122p::GFP 20 ng/ul]* | *egl-6* overexpression with *egl-6* promoter driving *egl-6* genomic sequence (gs) in *flp-6* mutants (*flp-6* bypass through *egl-6* overexpression).  Fig. 4I, Supplementary Fig. 10D |
| DAG1505 | *domEx1505 [mec-3p::QF 20 ng/ul, QUASp::TeTxcds::SL2mcherry 40 ng/ul, unc122p::GFP 20 ng/ul]* | Expression of Tetnus toxin in *mec-3* cells.  Fig. 5B, Supplementary Fig. 11B |
| AQ2145 | *ljEx19[egl-46p::YC2.3; lin15(+)]* | Camelon in FLP (Gift from Bill Schafer) ^89^.  Fig. 5C-E |
| DAG356 | *domIs355 [mec-3p::QF, mec-4p::QS, QUAS::CoChR::GFP, unc122p::RFP]* | [FLP::CoChR] FLP optogenetic background.  Fig. 5-7 |
| CB1112 | *cat-2(e1112) II* | Obtained from CGC.  Fig. 6A, Supplementary Fig. 12A |
| DAG1079-1080 | *eat-4(dom15) III; domIs355; domEx1079-1080 [mec-3p_eat-4CDS,SL2mCherry_ unc122p::GFP]* | Rescue of *eat-4* mutation with *mec-3* promoter driving *eat-4* coding sequence (cds)  Fig. 6E, Supplementary Fig. 12B |
| DAG1556 | *domIs355; cat-2(e1112) II* | *cat-2* mutant in FLP optogenetic background. Fig. 6B-D |
| DAG411 | *domIs355; eat-4(ky5) III* | *eat-4* mutant in FLP optogenetic background.  Fig. 6F-H |
| RB982 | *flp-21(ok889) V* | Obtained from CGC.  Fig. 7A, Supplementary Fig. 14A |
| tm1880 | *nlp-14(tm1880) X* | Obtained from NBRP Japan.  Fig. 7A, Supplementary Fig. 14A |
| tm2427 | *flp-13(tm2427) IV* | Obtained from NBRP Japan.  Fig. 7A, Supplementary Fig. 14A |
| tm12132 | *flp-5(tm12132) X* | Obtained from NBRP Japan.  Fig. 7, Supplementary Fig. 14, 13 |
| DAG1400 | *domIs355; flp-5(tm12132) X* | *flp-5* mutant in FLP optogenetic background.  Fig. 7B-D |
| DAG1558 | *domIs355; egl-3(gk238) V* | *egl-3* mutant in FLP optogenetic background.  Fig. 7B-D |
| DAG1506-1507 | *domEx1506-1507 [flp-5p::QF 30 ng/ul, QUASp::flp-5cds::SL2mcherry 30 ng/ul, unc122p::GFP 20 ng/ul]* | *flp-5* overexpression with *flp-5* promoter driving *flp-5* coding sequence (cds).  Fig. 7E, Supplementary Fig. 14B |
| DAG1683-1684 | *flp-5(tm12132) X; domEx1683-1684 [mec-3p::QF 30 ng/ul, QUASp::flp-5cds::SL2mcherry 30 ng/ul, unc122p::GFP 20 ng/ul]* | *flp-5* rescue with *mec-3* promoter driving *flp-5* coding sequence (cds).  Fig. 7E, Supplementary Fig. 14B |
| DAG1508-1509 | *domEx1508-1509 [mec-3p::QF 20 ng/ul, QUASp::flp-5cds::SL2mcherry 20 ng/ul, unc122p::GFP 20 ng/ul]* | *flp-5* overexpression with mec-3 promoter driving flp-5 coding sequence (cds).  Fig. 7E, Supplementary Fig. 14B |
| DAG1510-1511 | *dmsr-1(qn45) V; domEx1510-1511 [mec-3p::QF 20 ng/ul, QUASp::flp-5cds::SL2mcherry 20 ng/ul, unc122p::GFP 20 ng/ul]* | *flp-5* overexpression with *mec-3* promoter driving *flp-5* coding sequence (cds) in *dmsr-1* mutant background.  Fig. 7H, Supplementary Fig. 14C |
| DAG1568 | *flp-5(tm12132) X; dmsr-1(qn45) V* | *flp-5; dmsr-1* double mutant.  Fig. 7H-J, Supplementary Fig. 14C |
| MT1222 | *egl-6(n592) X* | Obtained from CGC.  Fig. 7K, Supplementary Fig. 14D |
| DAG1514-1515 | *domEx1514-1515 [egl-6p::QF 30 ng/ul, QUASp::egl-6gs::SL2mcherry 30 ng/ul, unc122p::GFP 20 ng/ul]* | *egl-6* overexpression with *egl-6* promoter driving *egl-6* genomic sequence (gs)*.*  Fig. 7K, Supplementary Fig. 14D |
| DAG1565-1567 | *domEx1565-1567 [egl-6p::QF 30 ng/ul, QUASp::dmsr-7cds::SL2mcherry 30 ng/ul, unc122p::GFP 20 ng/ul]* | *dmsr-7* overexpression with *egl-6* promoter driving *dmsr-7* coding sequence (cds).  Fig. 7K, Supplementary Fig. 14D |
| DAG1561-1562 | *domEx1561-1562 [egl-6p::QF 30 ng/ul, QUASp::dmsr-1Acds::SL2mcherry 30 ng/ul, unc122p::GFP 20 ng/ul]* | *dmsr-1A* overexpression with *egl-6* promoter driving *dmsr-1A* coding sequence (cds).  Fig. 7K, Supplementary Fig. 14D |
| DAG1563-1564 | *domEx1563-1564 [egl-6p::QF 30 ng/ul, QUASp::dmsr-1Bcds::SL2mcherry 30 ng/ul, unc122p::GFP 20 ng/ul]* | *dmsr-1B* overexpression with *egl-6* promoter driving *dmsr-1B* coding sequence (cds).  Fig7K, Supplementary Fig. 14D |
| DAG1520-1521 | *domEx1520-1521 [dmsr-1p::QF 20 ng/ul, QUASp::dmsr-1Acds::SL2mcherry 20 ng/ul, unc122p::GFP 20 ng/ul]* | *dmsr-1A* overexpression with *dmsr-1* promoter driving *dmsr-1A* coding sequence (cds).  Fig. 7L, Supplementary Fig. 14E |
| DAG1595-1596 | *domEx1595-1596 [lgc-39p::QF 30 ng/ul, QUASp::dmsr-1Acds::SL2mcherry 20 ng/ul, unc122p::GFP 20 ng/ul]* | *dmsr-1A* overexpression with *lgc-39* promoter driving *dmsr-1A* coding sequence (cds).  Fig. 7L, Supplementary Fig. 14E |
| DAG1805 | *domEx1805[ttx-1p::QF 20 ng/ul, QUASp::YC2.3(D21A, D57A, D94A, D130A) 60ng/ul, unc122p::RFP 20 ng/ul]* | Expression of YC2.3(D21A, D57A, D94A, D130A) cameleon in AFD.  Supplementary Fig. 10E |
| DAG1811 | *domEx1811[mec-3p::QF 20 ng/ul,* *QUASp::YC2.3(D21A, D57A, D94A, D130A) 20ng/ul, unc122p::RFP 20 ng/ul]* | Expression of YC2.3(D21A, D57A, D94A, D130A) cameleon in FLP.  Supplementary Fig. 11C |
